# *In vitro* activity of novel apramycin-dextran nanoparticles and free apramycin against selected Dutch and Pakistani *Klebsiella pneumonia* isolates

**DOI:** 10.1016/j.heliyon.2023.e22821

**Published:** 2023-11-25

**Authors:** Nagina Atlas, Bushra Uzair, Julie Movellan, Raquel Gracia, Damien Dupin, Iraida Loinaz, Cornelus F. van Nostrum, John P. Hays

**Affiliations:** a Dept. Biological Science, International Islamic University Islamabad, Pakistan; b Utrecht Institute for Pharmaceutical Sciences, Dept. of Pharmaceutics, Utrecht University, Utrecht, the Netherlands; c CIDETEC, Basque Research and Technology Alliance (BRTA), Parque Científico y Tecnológico de Gipuzkoa, Miramon Pasealekua, 196, Donostia-San Sebastián 20014, Spain; d Dept. Medical Microbiology & Infectious Diseases, Erasmus University Medical Centre (Erasmus MC), Rotterdam, the Netherlands

**Keywords:** Apramycin, Aminoglycoside, Dextran nanoparticles, *Klebsiella pneumoniae,* antimicrobial resistance

## Abstract

*Klebsiella pneumoniae* are bacteria associated with respiratory tract infections and are increasingly becoming resistant to antibiotics, including carbapenems. Apramycin is a veterinary antibiotic that may have the potential to be re-purposed for use in human health, for example, for the treatment of respiratory tract infections after coupling to inhalable nanoparticles. In the present study, the antibiotic apramycin was formulated with single chain polymeric nanoparticles and tested in free and formulated forms against a set of 13 *Klebsiella pneumoniae* isolates (from the Netherlands and Pakistan) expressing different aminoglycoside resistance phenotypes. Minimum Inhibitory Concentration, Time Kill Kinetics and biofilm experiments were performed providing evidence for the potential efficacy of apramycin and apramycin-based nanomedicines for the treatment of human *Klebsiella pneumonia* infections.

## 1 Background

*Klebsiella pneumoniae* is a Gram-negative bacterial member of the Enterobacteriaceae family that is frequently associated with catheter-associated urinary tract infection and lower respiratory tract infections [1]. Unfortunately, the extensive use of antibiotics has resulted in the isolation of multi-drug resistant *K. pneumoniae*, which is linked to high patient mortality rates [[2], [3], [4], [5], [6]]. However, the use of combinations of antibiotics, including the use of aminoglycosides, have been shown to be potentially effective against carbapenem resistant *K. pneumoniae* [7], of which plazomicin and apramycin may be most effective [8]. Additionally, *K. pneumoniae* is capable of creating biofilms - bacterial communities that adhere to surfaces and have reduced metabolic activity – resulting in increased resistance to antibiotics [9]. Therefore, new treatment strategies are ultimately required to treat both multidrug resistant and biofilm forms of *K. pneumoniae* strains [10].

Apramycin is a veterinary aminoglycoside antibiotic, potentially useful in humans, that is not inactivated by many of the aminoglycoside modifying enzymes found in human bacterial infections [[11], [12], [13], [14]]. Another useful characteristic of apramycin is that it appears to have fewer ototoxic and nephrotoxic side effects [[15], [16], [17], [18]] and phase I study trials of apramycin (EBL-1003) have reported that the drug in healthy volunteers is “safe and well tolerated” (https://www.swissbiotech.org/listing/juvabis-positive-phase-i-results-for-infection-treatment-caused-by-multidrug-resistant-bacteria/). From previous publications, apramycin has been shown to have antibacterial activity against a range of bacterial species [[19], [20], [21], [22], [23], [24]]. For example, a study of Enterobacteriaceae showed that 31 out of 31 meropenem susceptible *K. pneumoniae* isolates showed apramycin MICs of <16 mg/L. However, 14 out of 44 meropenem resistant *K. pneumoniae* isolates exhibited apramycin MICs of >256 mg/L [25] and more recently apramycin resistance was reported in an epidemic *K. pneumoniae* ST258 clone [26].

The targetting of antibiotics to the actual site of infection has the potential to reduce side effects and increase the efficacy of antibiotic therapy. Antibiotic-loaded nanoparticles (nanoantibiotics) are particularly interesting, as they could be inhaled as aerosols by patients suffering from respiratory tract infections [27]. The nanoparticle may consist of different forms, including lipid-based, inorganic, or polymeric-based nanoparticles [28]. It has been shown that the size and charge of nanoparticles are key for their interaction with mucus and extracellular matrix. For example, small nanoparticles (<500 nm in diameter) with negative or neutral surface charge can diffuse more efficiently through the mucus layer because they are less likely to be captured by eDNA in the EPS [[29], [30], [31], [32], [33]].

Single chain polymeric nanoparticles that are based on naturally occurring dextran polysaccharides (DXT-SCPN) are effective in loading and delivering antibiotics and antimicrobial peptides (https://patents.google.com/patent/WO2016071258A1/en) [34]. DXT-SCPNs are obtained by the controlled collapse of a single dextran chain, resulting in the formation of a small nanoparticle with sizes mainly reported as below 20 nm [[35], [36], [37], [38], [39], [40]]. Further, due to their small hydrodynamic diameter, a homogenous lung distribution can be observed (using SPECT-CT) after intra-tracheal administration using a Penn Century Microsprayer® aerosolizer, specifically indicating that DXT-SCPNs may be suitable nanocarriers for antibiotic delivery to the lung [34,[41], [42], [43]]. In addition, negatively-charged DXT-SCPNs can be formulated with high amount of cationic antibiotic, at least 20 wt%, via electrostatic interactions and the resulting nanoantibiotic exhibit a neutral charge, as shown by aqueous electrophoresis studies. In previous work, up to 40 wt% of tobramycin was loaded in DXT-SCPNs and formulated using the DNase I enzyme. These nanoformulations were then tested against mature continuous-flow *P. aeruginosa* biofilms, showing a better efficacy as compared to the free soluble treatment, tobramycin and DNase I [44].

In this publication, the authors aim was to determine the killing efficiency and biofilm formation inhibition of free apramycin versus dextran polysaccharide-apramycin nanoparticles for the bacterial pathogen *Klebsiella pneumoniae*. We show the results of *in vitro* antibiotic sensitivity profiling of apramycin formulated with DXT-SCPN nanoparticles (DXT-SCPN-Apra) and free apramycin (Apra) against *K. pneumoniae* isolates expressing different aminoglycoside sensitivity profiles and from two different countries (the Netherlands and Pakistan). Experiments were also performed to determine the efficacy of DXT-SCPN-Apra on biofilm formation of selected isolates. To the best of our knowledge this is the first work investigating the efficacy of apramycin loaded nanoparticles against planktonic and biofilm-producing *K. pneumoniae*.

## 2 Methods

### 2.1 Synthesis of DXT-SCPN

DXT-SCPN were prepared as previously described [42]. Briefly, dextran methacrylate (DXT-MA) was dispersed into phosphate-buffered saline (PBS) at 10 mg/ml, and the pH adjusted to 9 using a solution of sodium hydroxide (NaOH). A dithiol cross-linker, DODT, was added at a controlled rate using a syringe pump to cross-link the individual polymer chains to form DXT-SCPN. To obtain negatively charged DXT-SCPNs, the remaining methacrylate groups were quenched using mercaptopropionic acid. The resulting nanoparticles were then purified using dialysis until the conductivity of the dialysis water was below 1 μS/cm. Yield >90 %. DS = 38 %, CL = 5 %, ^1^H NMR (Bruker AVANCE III spectrometer, 500 MHz, D_2_O set at 4.79 ppm) *δ* ppm: 5.45–4.90 (3.5H, including H-1 and substituted H-2/3), 4.27–3.34 (14.7H, m, rest of Glc units and CH_2_O of cross-linker), 3.00–2.68 (4.9H, m, CH(CH_3_)CH_2_S, CH_2_S of cross-linker- and MPA-containing Glc units), 2.53–2.42 (1.7H, m, CH_2_COOH), 1.39–1.13 (3H, CH_3_ of cross-linker- and MPA-containing Glc units); Mw (GPC) = 86 kDa, Mw/Mn = 1.59 (calibration with polysaccharides standards); Dh (DLS) = 14 ± 1 nm; PDI 0.22, Zeta potential (pH = 7.2, 25 °C) = −23 mV ± 2.

### 2.2 Preparation of DXT-SCPN-Apra nanoantibiotic

**Fig. 1:**
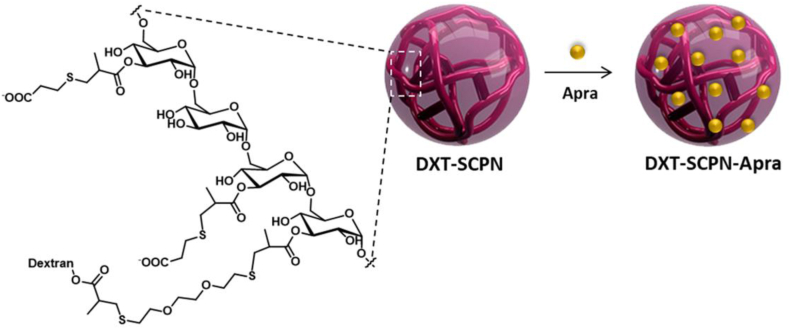
Schematic representation of negatively charged dextran nanoparticles (DXT-SCPN) and its formulation with apramycin (Apra) to form apramycin nanoantibiotic (DXT-SCPN-Apra).

The nanoantibiotic, DXT-SCPN-Apra was prepared by mixing 70 mg of DXT-SCPNs at pH 7 in water at 10 mg/ml with 3 mg of apramycin at pH 7 at 10 mg/ml in water. This mixture was stirred for 1 h at room temperature. Finally, the formulation was freeze-dried to obtain the nanoantibiotic formulation as a dried powder to be reconstituted before use (Fig. 1).

### 2.3 Dynamic Light Scattering (DLS) and aqueous electrophoresis studies

DLS analyses were conducted using a Zetasizer Nano ZS, ZEN3600 Model (Malvern Instruments Ltd) to obtain the hydrodynamic diameter of DXT-SCPNs and DXT-SCPN-Apra nanoantibiotic. All measurements were performed in disposable sizing cuvettes using a laser wavelength of 633 nm and a scattering angle of 173°. Aqueous electrophoresis was used to determine the Zeta-potential and the measurements were performed in disposable zeta potential cells (pH 7.4, 25 °C). For measurement, the samples were distributed in PBS solution for hydrodynamic diameter measurements or in 1 mM NaCl for Zeta-potential measurements (at a concentration of 10 mg/ml). The measurement of each sample was repeated three times at 25 °C.

### 2.4 Proton nuclear magnetic resonance (^1^H NMR)

^1^H NMR spectra were recorded on a Bruker AVANCE III spectrometer at 500 MHz and 25 °C in D_2_O. Chemical shifts (*δ*) were recorded as ppm relative to the residual signal of the solvent.

### 2.5 Bacterial isolates

A collection of eighteen *K. pneumoniae* was used (comprising the isolates ATCC 13883, B-DYK 9557 (blood), B-DYK 12747 (blood), ESBL-635 (rectum), ESBL-1059 (rectum), R-DYK 3427 (urine), R-DYK 4861(perineum), R-DYK 7926 (rectum) from the Netherlands, and U-546, U-675, U-716, T-467, T-521, T-876, B-137, B-432, B418, B-881 from Pakistan). Isolates from the Netherlands were obtained from the Erasmus University Medical Center Rotterdam (Erasmus MC) between 2010 and 2013. Isolates from Pakistan originated from various clinical sources i.e., blood (B-), urine (U-) and tracheal aspirate (T-), and were obtained from the Fouji Foundation Hospital Rawalpindi Pakistan between 2019 and 2020 (Table 1). Isolates were identified using the VITEK system (https://www.biomerieux-usa.com/vitek-2).

**Table 1:** Details of bacterial isolates used in this study.

Specimen Name	Country of Origin	Specimen Type	Date Isolated
ATCC 13883	Type strain		
B-DYK 9557	the Netherlands	Blood	04–2012
B-DYK 12747	the Netherlands	Blood	11–2012
ESBL 635	the Netherlands	Rectum	05–2012
ESBL 1059	the Netherlands	Rectum	12–2012
R-DYK 3427	the Netherlands	Urine	12–2010
R-DYK 4861	the Netherlands	Perineum	01–2012
R-DYK 7926	the Netherlands	Rectum	12–2013
U-546	Pakistan	Urine	04–2019
U-675	Pakistan	Urine	04–2019
U-716	Pakistan	Urine	01–2020
T-467	Pakistan	Throat	02–2020
T-521	Pakistan	Throat	02–2020
T-876	Pakistan	Throat	12–2019
B-137	Pakistan	Blood	11–2019
B-418	Pakistan	Blood	04–2019
B-432	Pakistan	Blood	04–2019
B-881	Pakistan	Blood	12–2019

### 2.6 Antimicrobial agents

Four aminoglycosides (Gentamicin, Tobramycin, Amikacin and Apramycin) were used in this study. Gentamicin and tobramycin ampules, as well as amikacin sulphate and apramycin sulphate were obtained from Sigma-Aldrich (the Netherlands). In Pakistan, gentamicin sulphate and amikacin sulphate ampules were purchased from Ray Pharma and tobramycin sulphate ampules were purchased from AGP pharma.

### 2.7 Aminoglycoside sensitivity profiling

The phenotypic susceptibilities of eighteen *K. pneumoniae* isolates (ATCC 13883, B-DYK 9557, B-DYK 12747, ESBL-635, ESBL-1059, R-DYK 3427, R-DYK 4861 R-DYK 7926 from the Netherlands, and U-546, U-675, U-716, T-467, T-521, T-876, B-137, B-432, B418, B-881 from Pakistan) against a range of aminoglycoside antibiotics was determined in duplicate using the micro broth dilution method of the European Committee on Antimicrobial Susceptibility Testing (https://www.eucast.org/fileadmin/src/media/PDFs/EUCAST_files/Breakpoint_tables/v_12.0_Breakpoint_Tables.pdf). For apramycin, phenotypic MIC values (sensitive, intermediate or resistant) were based on the National Antimicrobial Resistance Monitoring.

System (S, 8 mg/L; I, 16 or 32 mg/L; R, 64 mg/L) [20].

Stock solutions of aminoglycosides (gentamicin, tobramycin, amikacin and apramycin) were prepared by dissolving in distilled water to final concentrations of 512 mg/L. Serial dilutions (1: 2) of this stock solution were made from 512 to 0.125 mg/L using MH broth in a total volume of 50 μl per well in a 96-well microplate. Then, 50 μl of bacterial inoculum (final inoculum - 5 × 10^5^ CFU/ml) was added to each well of the microplate and incubated at 37 °C for 16–20 h. The final concentrations of the antibiotics ranged from 64 to 0.125 mg/L, respectively. The positive control contained broth mixed with bacterial inoculum, while the blank control contained only broth.

### 2.8 Time Kill Kinetics (mini-TKK) analysis

The therapeutic efficacy of free apramycin and complexed DXT-SCPN-Apra nanoparticles against thirteen *K. pneumoniae* isolates (ATCC 13883, B-DYK 9557 R-DYK 4861, U-675, T-521, U-716, B-137, T-876, U-546, B-432, B418, T-467, B-881) was assessed using mini-TKK analysis according to CLSI guidelines [45]. Concentrations of 0 (control), 2, 8 and 32 mg/L of apramycin in free form and in DXT-SCPN-Apra nanoparticles were tested against a concentration of 5 × 10^5^ CFU/mL *K. pneumoniae* in exponential growing phase for up to 24 h at 37 °C. Two hundred and 50 μL of bacterial suspension were removed at 0, 1, 2, 4, 6 and 24 h of incubation, and bacterial cells were pelleted and washed in phosphate buffered saline (PBS) twice. 10-fold serial dilutions were then made and 200 μl of these dilutions plated onto Mueller Hinton agar for incubation at 37 °C and colony counting the following day. All experiments were performed in triplicate.

### 2.9 Biofilm formation assay

Qualitative Assay - The antibiofilm activity of DXT-SCPN-Apra, empty nanoparticles (DXT-SCPN) and free Apramycin (Apra) was assessed qualitatively using a variation of the quantitative biofilm formation assay [46]. For this experiment, an overnight culture of the target bacteria in MH broth was diluted to 5 × 10^5^ CFU/ml. Fifteen microlitres of this bacterial suspension was added to separate glass tubes containing 2.9 ml of sterilized MH broth along with 100 μl DXT-SCPN-Apra, DXT-SCPN or Apra at concentrations ranging from 4, 8, 16, 32, 64 and 128 mg/L. Negative controls (without the addition of the bacterial suspension) and positive controls (without the addition of DXT-SCPN-Apra, DXT-SCPN and Apra) were also prepared. The tubes were then incubated for 24 h at 37 °C and after incubation, the broth culture was removed and washed twice with PBS. The test tubes were then stained with crystal violet dye (0.1 %) for 30 min and excess dye removed by gentle washing with deionized water. The tubes were dried, and biofilm formation was assessed by observing the presence/absence of a thin layer of blue stained biofilm on the test tube walls.

Quantitative Assay - The microtiter plate assay was used for quantitative estimation of biofilm formation using 96-well microtiter plates inoculated with 100 μl of MH broth, 50 μl of overnight bacterial culture (diluted to a final concentration of 5 × 10^5^ CFU/ml), and 50 μl of DXT-SCPN-Apra, DXT-SCPN or Apra (at concentrations of 4, 8, 16, 32, 64 and 128 mg/L) [46]. The total volume for each microtiter plate well was 200 μl. After 24 h incubation at 37 °C, the content of the wells (including non-adherent bacterial cells) were gently removed and the wells washed three times with PBS. Any biofilm was then fixed using sodium acetate, followed by 10 min of crystal violet staining (0.1 %). The stained biofilms were then washed with distilled water and allowed to dry. Next, 200 μl of 95 % ethanol was pipetted into each well in order to elute the crystal violet stain from the biofilm. Finally, ethanol was pipetted into a cuvette and the absorbance of the ethanol measured at 620 nm using an ELISA reader. Negative (sterile MH broth) and positive (MH broth containing *K. pneumoniae* isolates) controls were also used in the assay via sterile growth medium.

The percentage of biofilm inhibition was calculated using the following formula:

(%ofbiofilminhibition)=[1–OD_(620nm)ofK.pneumoniaeexposedDXT‐SCPN‐Apra(orApra)/

OD_(620nm)non‐exposedK.pneumoniaecontrol]×100.

### 2.10 Production of extracellular polymeric substances (EPS)

To investigate the effect of DXT-SCPN-Apra, DXT-SCPN and Apra on the production of extracellular polymeric substances (EPS) three *K. pneumoniae* isolates (U-675, B-881 and T-876) were selected. An overnight bacterial suspension in MH broth was diluted to a final concentration of 5 × 10^5^ CFU/ml and 2 ml was added to 100 ml of MH broth containing DXT-SCPN-Apra, Empty Nanoparticles or free Apramycin at sub-inhibitory concentrations. Based on previous MIC results (see Table 1.), 8 mg/L concentration of apramycin for DXT-SCPN-Apra or Apra was used with *K. pneumoniae* isolates B-881 and T-876, and 2 mg/L concentration of apramycin for DXT-SCPN-Apra or Apra was used with *K. pneumoniae* isolate U-675. Positive controls were prepared without the addition of DXT-SCPN-Apra, DXT-SCPN or Apra. All flasks were incubated for 24 h at 37 °C in a shaking incubator. EPS was extracted after incubation by centrifuging bacterial cultures at 6000 rpm for 30 min at 4 °C. The supernatant was then mixed with two volumes of acetone and the combination was chilled overnight (at 4 °C) to precipitate EPS. Finally, the EPS product was collected by centrifugation at 6000 rpm at 4 °C for 30 min. Both wet pellet weight and then dry pellet weight (after drying the wet pellet at 40 °C for 24 h) were measured [47]. Statistically significant differences between dry and wet pellet weights were calculated using the Students t-test.

## 3 Results

### 3.1 Preparation of DXT-SCPN-Apra nanoantibiotic

**Fig. 2:**
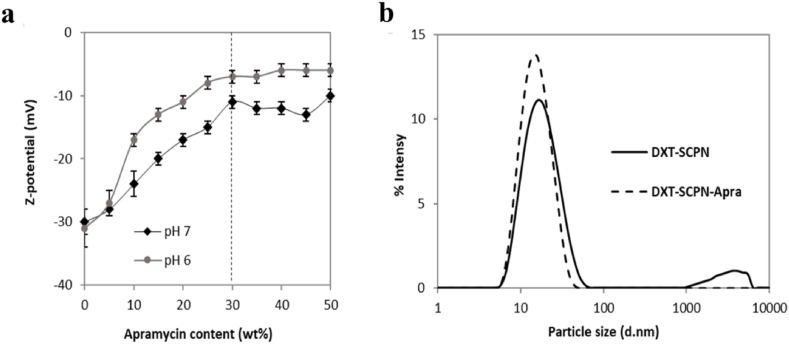
a) Variation of Zeta-potential DXT-SCPN-Apra formulation in function of the amount of apramycin (wt%) added as judged by aqueous electrophoresis at 25 °C in the presence of 1 mM of NaCl; b) Size distribution obtained by DLS at 10 mg/ml in PBS.

**Fig. 3:**
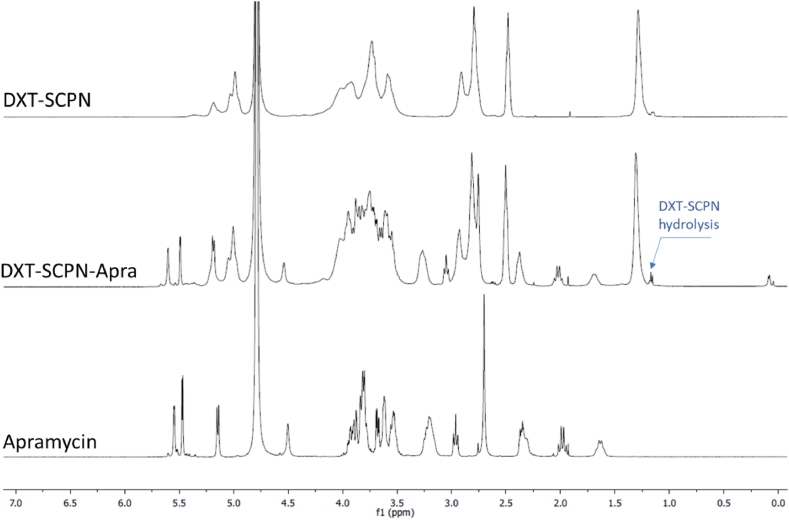
^1^H NMR spectra (D_2_O) of DXT-SCPN, DXT-SCPN-Apra and apramycin sulphate at pH 7.

DXT-SCPNs with a hydrodynamic diameter (D_h_) of 17 nm and a Zeta potential (Z-potential) of −31 mV were prepared via the controlled collapse of single branched polymer coil into nanoparticles through intrachain cross-linking [42]. The positively charged apramycin (pKa of the apramycin amine groups = 8.2, 6.6, 7.7, 7.5, 6.7), was electrostatically attached to the negatively charged DXT-SCPN (pKa = 4.9, experimentally determined) [17]. The electrostatic interactions between DXT-SCPN and apramycin were monitored by aqueous electrophoresis until reaching a Z-potential plateau from 30 wt% apramycin loading (Fig. 2a.). The increase of Z-potential observed ranged from −31 (±1) mV for DXT-SCPN, up to −12 (±1) mV for DXT-SCPN-Apra, which confirmed that both charged entities were neutralized by the shielding of the anionic nanocarrier by the cationic antibiotic via electrostatic interactions. Dynamic Light Scattering (DLS, Fig. 2b) was used to measure the hydrodynamic diameter of DXT-SCPN-Apra (D_h_ = 14 ± 1 nm) and confirmed the absence of aggregates during the formulation process, the size of the nanoantibiotic being slightly lower than the size of the nanocarrier (D_h_ = 17 ± 1 nm). ^1^H NMR (D_2_O) of DXT-SCPN-Apra nanoparticles at pH 7 (Fig. 3.) confirmed a loading of 30 % of apramycin and less than 5 % hydrolysis of the ester groups during the formulation process, which can be monitored by the integration of the peak at 1.17 ppm (<5 % hydrolysis). The amount of apramycin in the formulation was calculated by integrating the peak at 1.68 ppm corresponding to 1 proton of apramycin and the peak at 1.31 ppm corresponding to the 3 protons of the methyl groups of the nanoparticle.

### 3.2 Antimicrobial susceptibility of *Klebsiella pneumoniae* collection

The antimicrobial susceptibility of Dutch and Pakistani *K. pneumoniae* isolates against clinically relevant aminoglycoside antibiotics (gentamicin, tobramycin, amikacin and apramycin) are shown in Table 2. Despite the different (MDR) phenotypes tested (range of: gentamicin MIC 0.25 – ≥64 mg/L; tobramycin MIC 0.25 - ≥64 mg/L; amikacin MIC 1 - ≥64 mg/L), apramycin MIC values varied from 4 to 16 mg/L.

**Table 2:** Antibiotic sensitivities for 18 *Klebsiella pneumoniae* isolates (Netherlands and Pakistan) expressing different aminoglycoside antibiotic sensitivity profiles. All isolates were tested in duplicate. If MIC values differed, both results are shown. (S) = Sensitive. (R) = Resistant.

*Klebsiella pneumoniae* isolate	Aminoglyocside MIC (mg/L)			
	Gentamycin	Tobramycin	Amikacin	APRAMYCIN
ATCC 13883	0.25 (S)	0.25 (S)	1 (S)	4/8
U- 675	0.25 (S)	0.5 (S)	1 (S)	4
T-521	0.25 (S)	1/0.5 (S)	1 (S)	4
U-716	0.25/0.5 (S)	0.25/1 (S)	1 (S)	4
ESBL-635	0.25 (S)	16 (R)	8 (S)	8
B-DYK 9557	0.25/0.5 (S)	16 (R)	16/32 (R)	8/16
B-137	0.5 (S)	16 (R)	8/16 (R)	4
B-DYK 127477	0.5 (S)	32 (R)	32 (R)	4
T-876	0.5 (S)	32 (R)	16 (R)	16
U-546	0.5/1 (S)	32 (R)	32 (R)	16
B-418	1 (S)	32 (R)	16 (R)	8/16
ESBL-1059	1 (S)	32 (R)	32 (R)	8
R-DYK 3427	1 (S)	64 (R)	64 (R)	8
B-432	0.5/1 (S)	64 (R)	64/≥ 64 (R)	8
T-467	32 (R)	64/≥ 64 (R)	≥ 64 (R)	16/8
R-DYK 4861	≥ 64 (R)	≥ 64 (R)	64/≥ 64 (R)	16
R-DYK 7926	≥ 64 (R)	16 (R)	2 (S)	16
B-881	64/≥ 64 (R)	≥ 64 (R)	≥ 64 (R)	16

Using our set of clinical *K. pneumoniae* isolates, those isolates sensitive to gentamicin, tobramycin and amikacin were also found to be sensitive (≤8 mg/L) to apramycin. Isolates sensitive to gentamicin, but resistant to tobramycin and amikacin, generally exhibited intermediate sensitivity (16 or 32 mg/L) for apramycin. Isolates resistant to gentamicin, tobramycin and amikacin were also found to show intermediate resistance to apramycin. Dutch and Pakistani isolates with similar aminoglycoside resistance phenotypes showed similar apramycin MIC phenotypes.

### 3.3 Time Kill Kinetics (mini-TKK) analysis

**Fig. 4:**
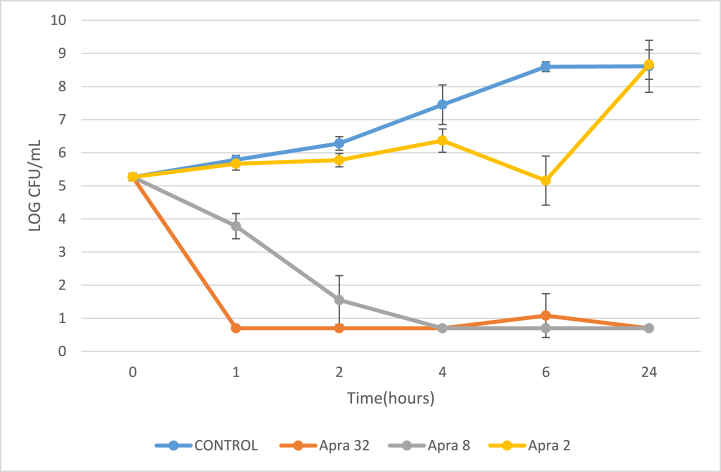
Time Kill Kinetics (TKK) of *K. pneumoniae* ATCC 13883 using free apramycin (Apra) at concentrations of 2, 8 and 32 mg/L. Shown here are the means of triplicate experiments with error bars indicating the standard deviation.

**Fig. 5:**
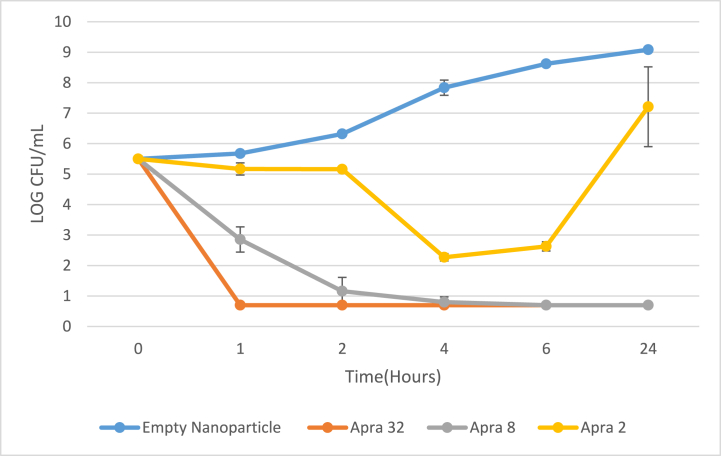
Time Kill Kinetics (TKK) of *K. pneumoniae* ATCC 13883 using DXT-SCPN-Apra at apramycin concentrations of 2, 8 and 32 mg/L. Shown here are the means of triplicate experiments with error bars indicating the standard deviation.

**Fig. 6:**
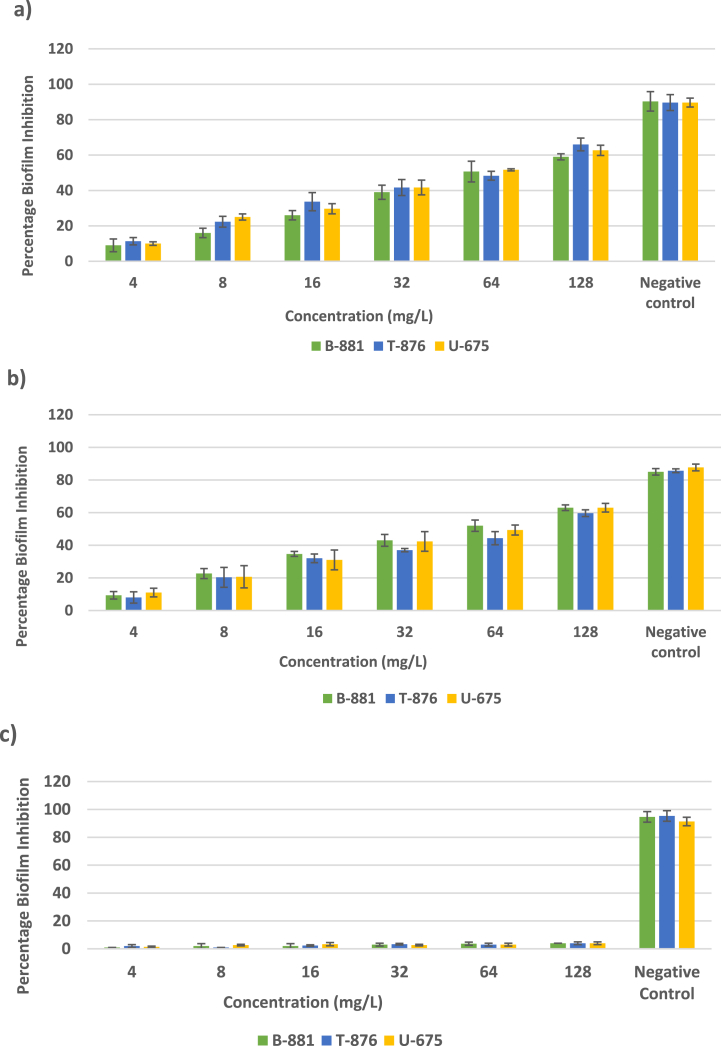
Percent inhibition of biofilm formation after exposure of *K. pneumoniae* isolates B-881, T-876 and U675 to various concentrations of a) DXT-SCPN-Apra, b) Apra and c) DXT-SCPN. The negative control contained sterile growth medium, and the positive control contained growth medium with *K. pneumoniae*.

**Fig. 7:**
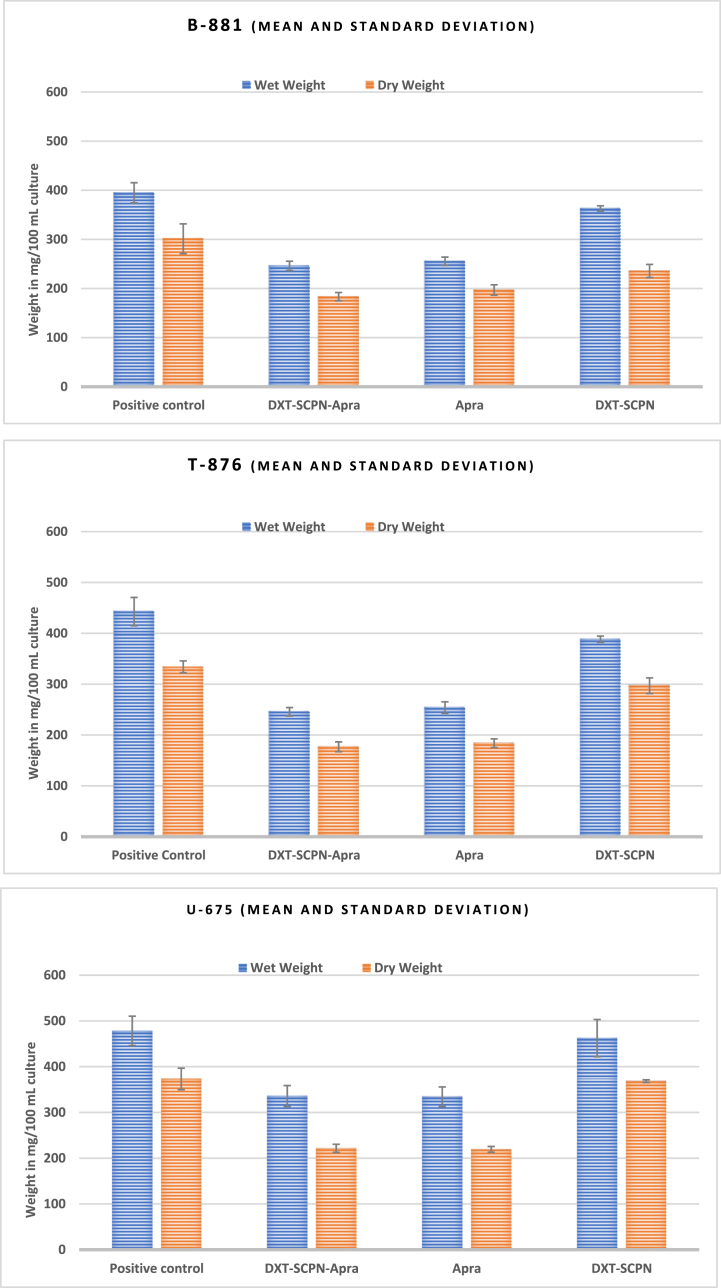
Quantification of EPS extracted from *K. pneumoniae* strains B-881, T-876 and U-675 in the presence or absence of DXT-SCPN-Apra, Apra and DXT-SCPN. The positive control contained growth medium and *K. pneumoniae* only.

The results using free apramycin (Apra) are presented in Fig. 4 and Supplementary Material-- Figs. 1–12. The results for apramycin nanoparticles (DXT-SDCPN-Apra) are presented in Fig. 5 and Supplementary Material - Figs. 13–24. With respect to free apramycin, the thirteen *K. pneumoniae* isolates tested generated no colony forming units after 6 h of exposure to 32 mg/L (limit of detection = 5 CFU/ml) and growth of all thirteen isolates remained suppressed for 24 h. At 2 mg/L concentration of free apramycin, bacteria grew up to the level of non-exposed bacteria observed after 24 h exposure. With respect to DXT-SCPN-Apra, the thirteen *K. pneumoniae* isolates tested did not generate colony forming units after 6 h of exposure to ≥8 mg/L. However, a small increase in growth was observed for 2 isolates after 24 h exposure to 8 mg/L and 32 mg/L DXT-SCPN-Apra. At 2 mg/L of DXT-SCPN-Apra, bacterial growth was initially suspended for 6 h, with bacterial re-growth occurring after 24 h.

### 3.4 Biofilm formation assay

Qualitative Assay - The effect of DXT-SCPN-Apra, DXT-SCPN and Apra on biofilm formation was estimated by the biofilm formation assay method. No visible biofilm formation was observed for both *K. pneumoniae* isolates B-881 and T-876 after treatment with DXT-SCPN-Apra and Apra at concentrations ≥32 mg/L, while in the case of strain U-675, no visible biofilm formation was observed at a concentration of ≥16 mg/L or above (Table 3, Table 4). As expected, treating *K. pneumoniae* isolates B-881, T-876 and U-675 with empty DXT-SCPN (at the concentrations of the nanocarrier corresponding to the one used for DXT-SCPN-Apra), had no effect on the biofilm formation, with biofilm formation being observed at all concentrations. (Table 5). Pictures of the reaction tubes are shown in Supplementary Materials - Figs. 25, 26 and 27.

**Table 3:** Effect of DXT-SCPN-Apra on biofilm formation. (-Clear), (+Slight), (++ Light), (+++ Medium), (++++ Strong).

Intensity of color of Biofilm	+ve control	-ve control	128 μg/ml	64 μg/ml	32 μg/ml	16 μg/ml	8 μg/ml	4 μg/ml
B-881	++++	_	_	_	_	+	+++	++++
T-876	++++	_	_	_	_	+	+++	++++
U-675	++++	_	_	_	_	_	+++	++++

**Table 4:** Effect of free apramycin (Apra) on biofilm formation. (-Clear), (+Slight), (++ Light), (+++ Medium), (++++ Strong).

Intensity of color of Biofilm	+ve control	-ve control	128 μg/ml	64 μg/ml	32 μg/ml	16 μg/ml	8 μg/ml	4 μg/ml
B-881	++++	_	_	_	_	+	+++	++++
T-876	++++	_	_	_	_	+	+++	++++
U-675	++++	_	_	_	_	_	++	++++

**Table 5:** Effect of empty nanoparticles (DXT-SCPN) on biofilm formation. (-Clear), (+Slight), (++ Light), (+++ Medium), (++++ Strong).

Intensity of color of Biofilm	+ve control	-ve control	128 μg/ml	64 μg/ml	32 μg/ml	16 μg/ml	8 μg/ml	4 μg/ml
B-881	++++	_	+++	+++	+++	++++	++++	++++
T-876	++++	_	++	+++	+++	++++	++++	++++
U-675	++++	_	++	+++	+++	++++	++++	++++

Quantitative Assay - In the majority of experiments, treatment of *K. pneumoniae* with sub-inhibitory concentrations of DXT-SCPN-Apra and Apra significantly reduced biofilm formation in a dose-dependent manner. As shown in Fig. 6, the percentage inhibition of biofilm formation was 59 % and 63 % for isolate B-881 when treated with DXT-SCPN-Apra and Apra at the highest concentration used (128 mg/L), while the percentage biofilm inhibition was 66 % and 59 % when isolate T-876 was used at 128 mg/L. For isolate U-675 the percentage inhibition at 128 mg/L was found to be 62 % and 63 %. DXT-SCPN nanoparticles without any apramycin had no significant effect on the biofilm inhibition (Fig. 6).

### 3.5 Production of extracellular polymeric substance (EPS)

To determine the effect of DXT-SCPN-Apra and Apra on the production of extracellular polymeric substances (EPS), *K. pneumoniae* strains B-881, T-876 and U-675 were grown in the presence/absence of sub-inhibitory concentrations of DXT-SCPN-Apra, Apra and DXT-SCPN. The results are shown in Fig. 7.

Wet Weight - A significant reduction in the amount of EPS (as wet weight) was observed between untreated *K. pneumoniae* bacteria and *K. pneumoniae* bacteria treated with DXT-SCPN-Apra (P < 0.05) and Apra (P < 0.05). No significant difference was observed between untreated *K. pneumoniae* bacteria and *K. pneumoniae* bacteria treated with DXT-SCPN (P > 0.05). For *K. pneumoniae* isolate B-881 treated with DXT-SCPN-Apra, Apra and DXT-SCPN, there was a 37.6 %, 35.3 % and 8 % decrease in the wet weight of EPS, respectively. For isolate T-876, the respective values were 44.5 %, 42.6 % and 12.3 %, whilst for isolate U-675, 29.8 %, 30 % and 3.3 %.

Dry Weight - A significant reduction in the amount of EPS (as dry weight) was observed between untreated *K. pneumoniae* bacteria and *K. pneumoniae* bacteria treated with DXT-SCPN-Apra (P < 0.05) and Apra (P < 0.05). No significant difference was observed between untreated *K. pneumoniae* bacteria and *K. pneumoniae* bacteria treated DXT-SCPN (P > 0.05). For *K. pneumoniae* isolate B-881 treated with DXT-SCPN-Apra, Apra and DXT-SCPN, there was a 39.2 %, 34.6 % and 21.8 % decrease in the wet weight of EPS, respectively. For isolate T-876, the respective values were 47 %, 45 % and 11 %, whilst for isolate U-675, 40.6 %, 41.3 % and 1.3 %.

## 4 Discussion

Apramycin was successfully formulated with DXT-SCPNs at 30 wt% and aqueous electrophoresis studies showed that the charges of both entities were compensated upon formulation as shown by the increase in zeta potential. The amount of loaded apramycin was found to be lower than that previously reported for DXT-SPCNs loaded with the aminoglycoside tobramycin and used to treat *P. aeruginosa* biofilms (40 wt%) [43], but higher than that previously obtained using antimicrobial peptides AA139 and M33, which reached only 10 wt% [34] and 14 wt% [44], respectively. Such difference can be explained by the differences in amine pKa between the antimicrobial compounds and antibiotics. For example, apramycin possesses slightly lower pKa than tobramycin, which results in a lower cationic charge at neutral pH and thus, has fewer positive groups for electrostatic interactions with the negatively charged DXT-SCPNs.

Results from MIC testing indicated that apramycin was effective against a range of aminoglycoside sensitive and resistant *K. pneumoniae* isolates at MICs ranging from 4 to 16 mg/L. The potential significance of this finding is that aminoglycoside antibiotics (including apramycin) could be used to treat carbapenem resistant *K. pneumoniae* (CR-Kp). For example, Smith and Kirby (2016) investigated 141 Enterobacteriaceae, including 75 *K. pneumoniae* isolates, of which 44 were meropenem resistant (CR-Kp). Their conclusion was that among carbapenem-resistant Enterobacteriacea, 70.8 % were apramycin susceptible, which was favorable when compared to other aminoglycosides in clinical use [25]. Interestingly, combinations of apramycin with meropenem have indicated a synergistic effect against carbapenemase-producing and XDR *K. pneumoniae* strains [48]. This indicates that apramycin may be particularly successful if used in combination with other antibiotics.

The potential use of nanoparticle-formulated antibiotics to more efficiently treated antimicrobial resistant infections has been previously described [27,28], including the use of nanostructured aminoglycosides [49]. Therefore, after investigating the potential use of free apramycin (Apra) against a range of aminoglycoside resistant *K. pneumoniae* isolates, we investigated Apra and apramycin formulated with nanoparticles (DXT-SCPN-Apra) in TKK assays. For all 13 *K. pneumoniae* isolates tested, the use of Apra at 8 and 32 mg/L resulted in <10 CFU/ml between 1 and 6 h after exposure and no re-growth after 24 h. Similar results were obtained for DXT-SCPN-Apra, except for 2 isolates where re-growth of approximately 100 CFU/ml was observed after 24 h of culture. Any re-growth could theoretically be associated with the ability of certain isolates to recover from translational misreading of their RNA or from the cell membrane effects of aminoglycosides (https://en.wikipedia.org/wiki/Aminoglycoside). In any case, our results indicated that DXT-SCPN-Apra was just as effective as Apra at limiting *K. pneumoniae* growth within the first 6 h of bacterial exposure.

Biofilm-forming microbes can cause a variety of diseases, and according to one of the National Institutes of Health and Centre of Disease Control reports, biofilm-related microorganisms are associated with 65–80% of infections [50]. Using antibiotics to control the formation of biofilms could be an effective way to prevent many microbial infections. Therefore, the authors investigated the effects of DXT-SCPN-Apra and Apra on biofilm formation and extracellular polymeric substance (EPS) production in *K. pneumoniae*. EPS is synthesized by bacteria and utilized to establish the structure of biofilms, contributing approximately 50 %–90 % of the organic component present in biofilms (https://en.wikipedia.org/wiki/Extracellular_polymeric_substance). Importantly, interactions between biofilm EPS and nanoparticles (including antibiotic formulated with nanoparticles) could enhance antibiotic transport to the actual site of infection [51]. From our results using the qualitative biofilm assay and 3 *K. pneumoniae* isolates from blood, throat and urine, both Apra and DXT-SCPN-Apra inhibited biofilm formation at 32 mg/L (isolates B-881 and T-876) or 16 mg/L (isolate U-675). Using a quantitative biofilm assay these MICs translated into an approximate 40 % and reduction in biofilm formation (quantitative biofilm assay). Although the qualitative and quantitative biofilm assays have different levels of sensitivity and limit of detection, from the results it appears that apramycin has the ability to prevent biofilm formation in *K. pneumoniae* isolates that are sensitive (U-675) or resistant to gentamicin (B-881), tobramycin (T-876 and B-881) and amikacin (T-876 and B-881).

With respect to EPS production, after exposure to sub-inhibitory Apra and DXT-SCPN-Apra concentrations of 8 mg/L (isolates B-881 and T-876) and 2 mg/L (isolate U-675) was reduced by approximately 50 % (mg wet weight and mg dry weight) for all 3 isolates. As observed with biofilm formation *per se*, our results indicate that apramycin has the ability to reduce EPS formation (wet weight and dry weight) in *K. pneumoniae* isolates that are sensitive (U-675) or resistant to gentamicin (B-881), tobramycin (T-876 and B-881) and amikacin (T-876 and B-881). Finally, it is important to note that several different mechanisms for phenotypic aminoglycoside resistance exist, including aminoglycoside-modifying enzymes (AMEs) [11] and 16 S ribosomal methyl transferases [14]. Also, apramycin resistant clones of *K. pneumoniae* (e.g. ST258) [26] and resistance mechanisms (*Escherichia coli aac(3)-IV* gene present on a mobilisable plasmid) have been described [52]. However, although this was a pilot study and no *in vivo* work was performed, when taken together, our findings provide evidence for the potential efficacy of apramycin and apramycin-nanoparticles for the treatment of *K. pneumoniae* infections.

## Credit author statement

Damien Dupin: Writing – review & editing, Resources, Methodology, Investigation, Formal analysis. John Hays: Writing – review & editing, Writing – original draft, Supervision, Resources, Project administration, Methodology, Investigation, Formal analysis, Data curation, Conceptualization. Iraida Loinaz: Writing – review & editing, Supervision, Resources, Project administration, Methodology, Investigation, Funding acquisition, Formal analysis, Data curation. Cornelius F. van Nostrum: Writing – review & editing, Supervision, Resources, Project administration, Investigation, Funding acquisition, Conceptualization. Nagina Atlas: Writing – review & editing, Writing – original draft, Resources, Methodology, Investigation, Formal analysis, Conceptualization. Bushra Uzair: Writing – review & editing, Supervision, Resources, Project administration, Methodology, Funding acquisition, Conceptualization. Julie Movellan: Writing – review & editing, Methodology, Investigation. Raquel Gracia: Writing – review & editing, Resources, Methodology, Investigation

## Data avilabiliy

The data included in this article are referenced in the article or available in the supplemnatry data.

## Declaration of competing interest

The authors declare the following financial interests/personal relationships which may be considered as potential competing interests: Nagina Atlas, Bushra Uzair, Cornelus F. van Nostrum reports financial support was provided by the Higher Education Commission Pakistan. Julie Movellan, Raquel Gracia, Damien Dupin, Iraida Loinaz reports financial support was provided by the 10.13039/501100003086Basque Government.
