# Supplementary material for: In vitro activity of novel apramycin-dextran nanoparticles and free apramycin against selected Dutch and Pakistani Klebsiella pneumonia isolates
Source: Heliyon. 2023 Nov 25;9(12):e22821. doi: 10.1016/j.heliyon.2023.e22821 (PMC10730580; doi:10.1016/j.heliyon.2023.e22821)
Supplement: Multimedia component 1 [file mmc1.pdf]

Supplementary Material

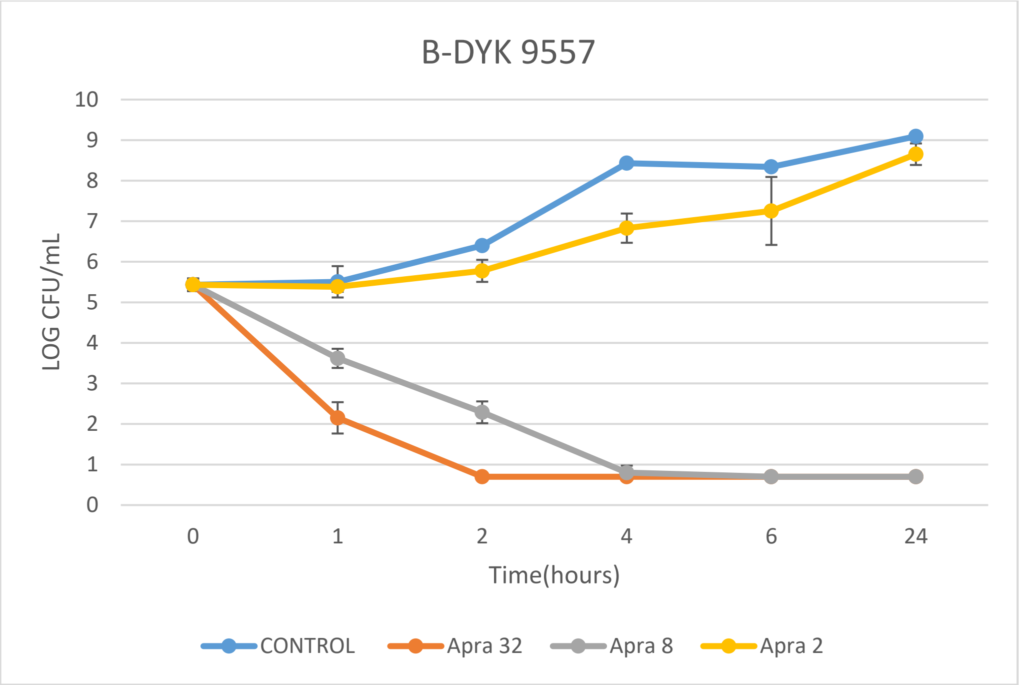

Supplementary Material - Figure 1. Time Kill Kinetics (TKK) of *K. pneumoniae* B-DYK 9557 using free apramycin (Apra) at concentrations of 2, 8 and 32 mg/L. Shown here are the means of triplicate experiments with error bars indicating the standard deviation.

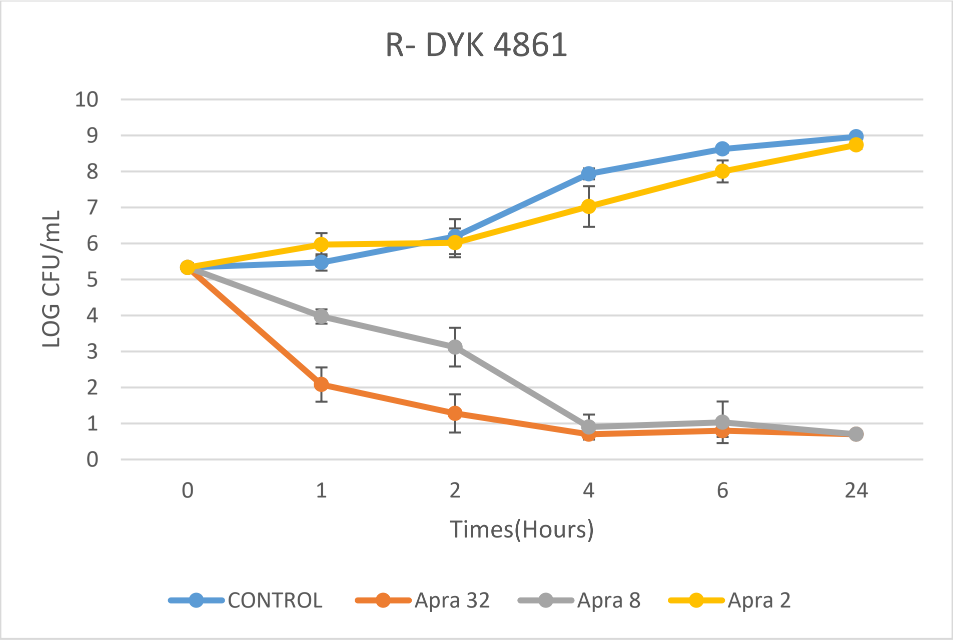

Supplementary Material - Figure 2. Time Kill Kinetics (TKK) of *K. pneumoniae* R-DYK 4861 using free apramycin (Apra) at concentrations of 2, 8 and 32 mg/L. Shown here are the means of triplicate experiments with error bars indicating the standard deviation.

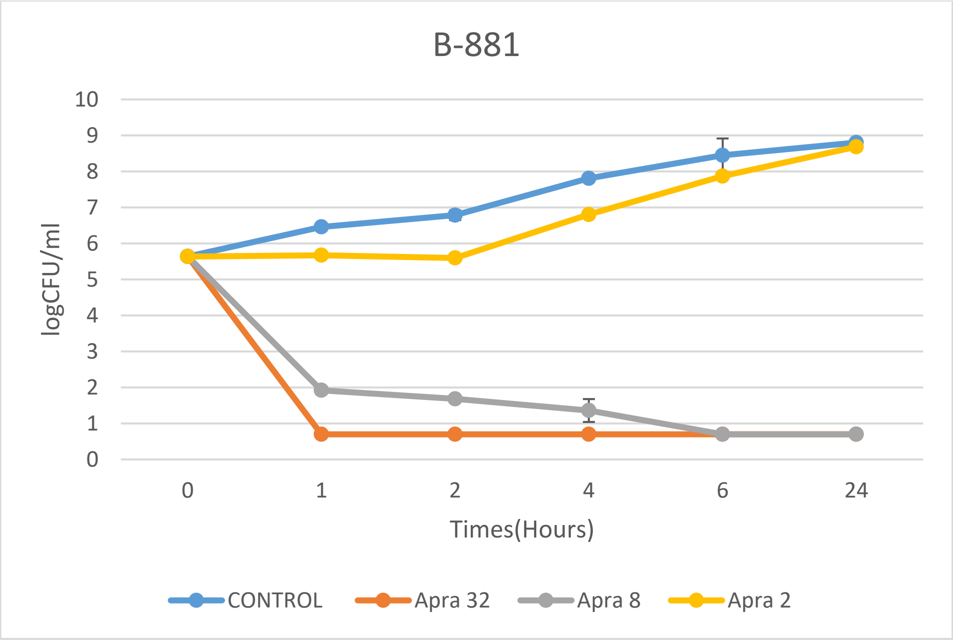

Supplementary Material - Figure 3. Time Kill Kinetics (TKK) of *K. pneumoniae* B-881 using free apramycin (Apra) at concentrations of 2, 8 and 32 mg/L. Shown here are the means of triplicate experiments with error bars indicating the standard deviation.

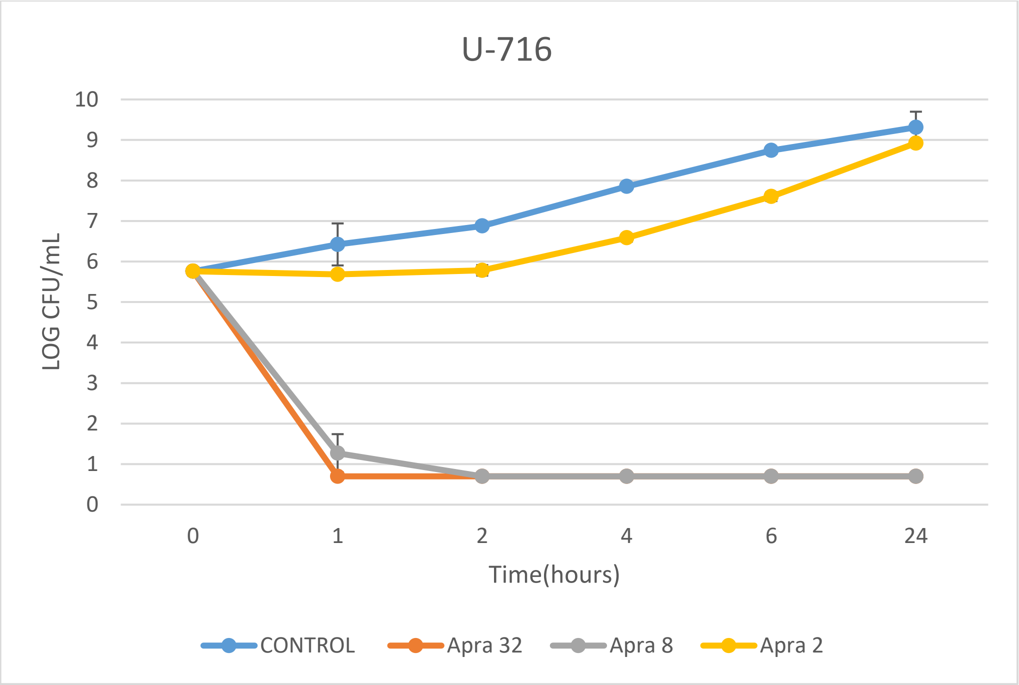

Supplementary Material - Figure 4. Time Kill Kinetics (TKK) of *K. pneumoniae* U-716 using free apramycin (Apra) at concentrations of 2, 8 and 32 mg/L. Shown here are the means of triplicate experiments with error bars indicating the standard deviation.

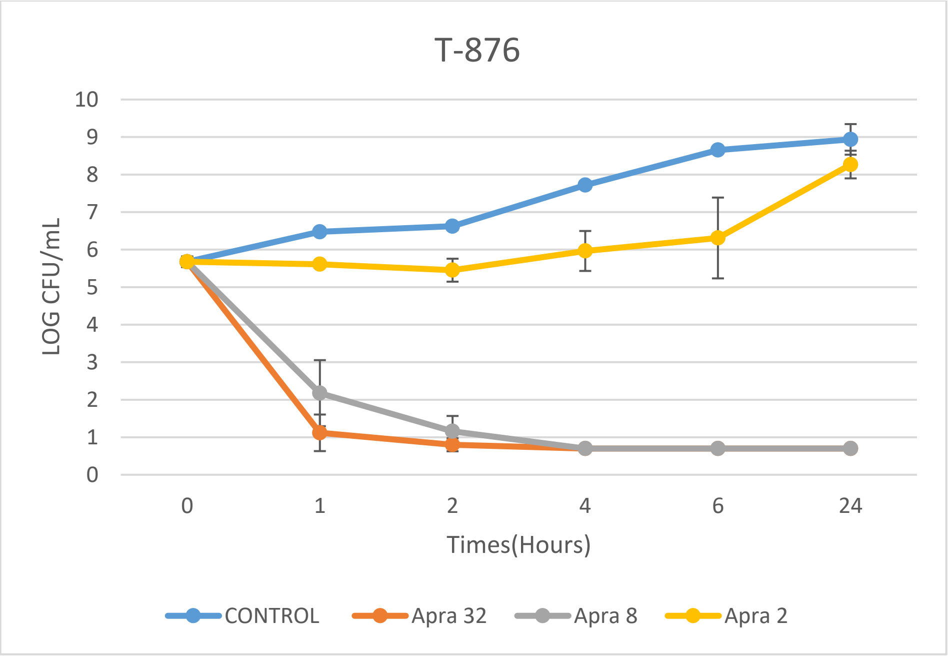

Supplementary Material - Figure 5. Time Kill Kinetics (TKK) of *K. pneumoniae* T-876 using free apramycin (Apra) at concentrations of 2, 8 and 32 mg/L. Shown here are the means of triplicate experiments with error bars indicating the standard deviation.

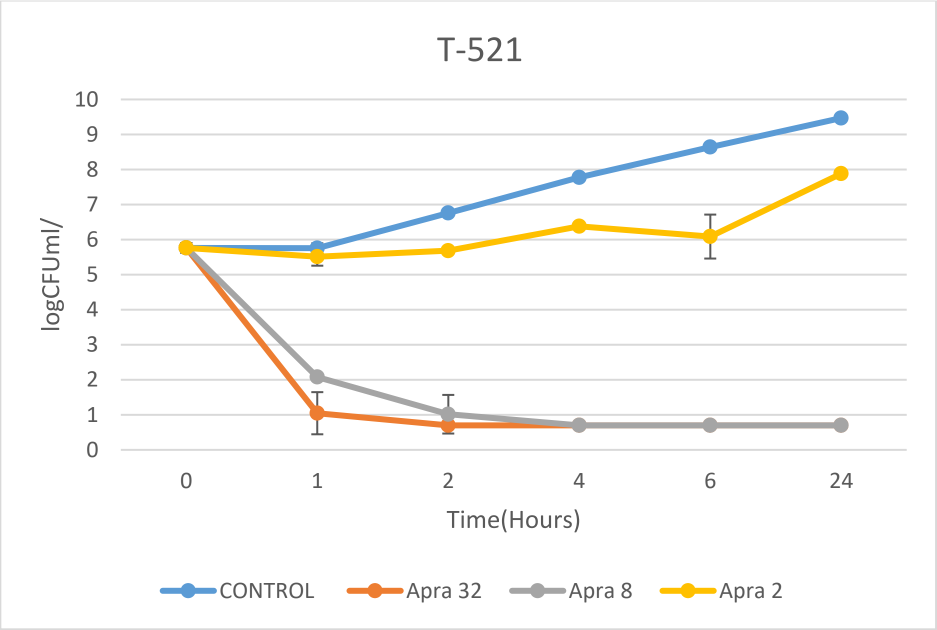

Supplementary Material - Figure 6. Time Kill Kinetics (TKK) of *K. pneumoniae* T-521 using free apramycin (Apra) at concentrations of 2, 8 and 32 mg/L. Shown here are the means of triplicate experiments with error bars indicating the standard deviation.

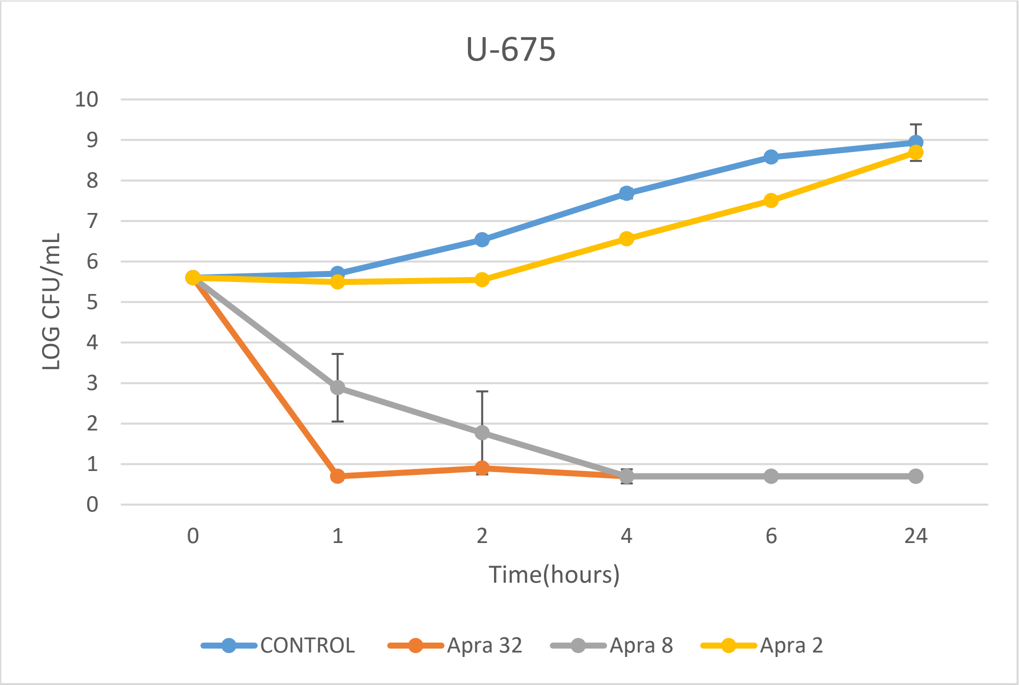

Supplementary Material - Figure 7. Time Kill Kinetics (TKK) of *K. pneumoniae* U-675 using free apramycin (Apra) at concentrations of 2, 8 and 32 mg/L. Shown here are the means of triplicate experiments with error bars indicating the standard deviation.

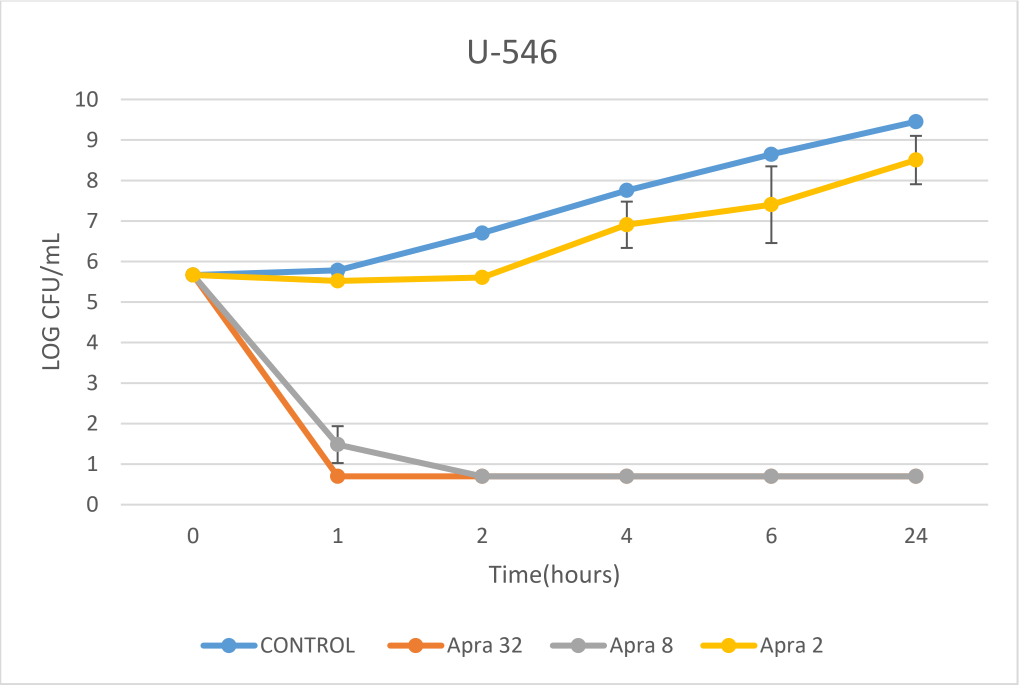

Supplementary Material - Figure 8. Time Kill Kinetics (TKK) of *K. pneumoniae* U-546 using free apramycin (Apra) at concentrations of 2, 8 and 32 mg/L. Shown here are the means of triplicate experiments with error bars indicating the standard deviation.

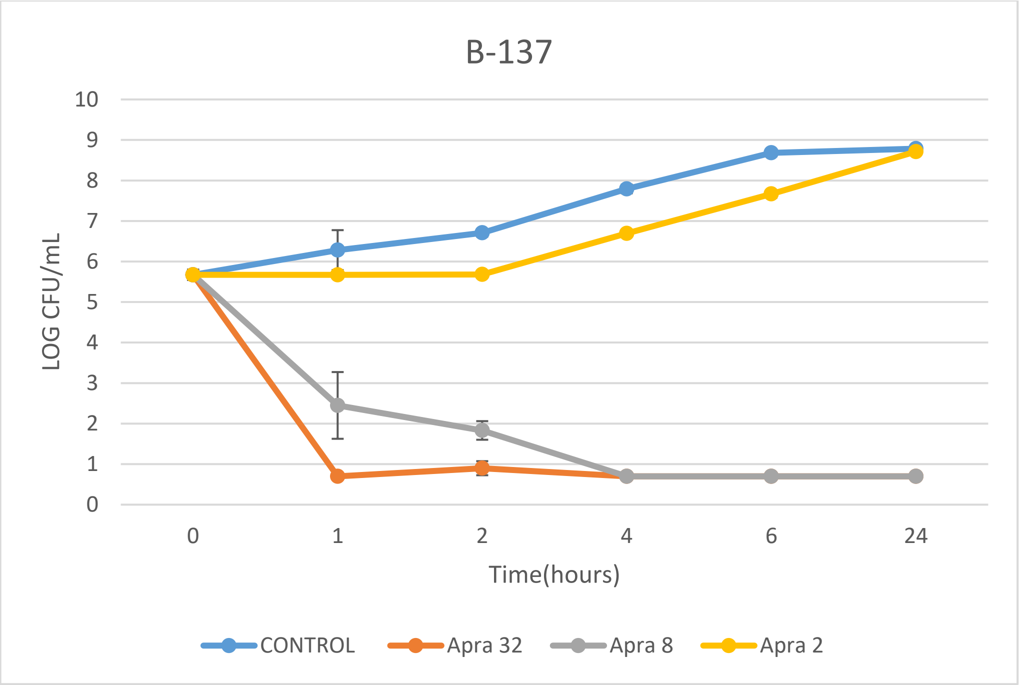

Supplementary Material - Figure 9. Time Kill Kinetics (TKK) of *K. pneumoniae* B-137 using free apramycin (Apra) at concentrations of 2, 8 and 32 mg/L. Shown here are the means of triplicate experiments with error bars indicating the standard deviation.

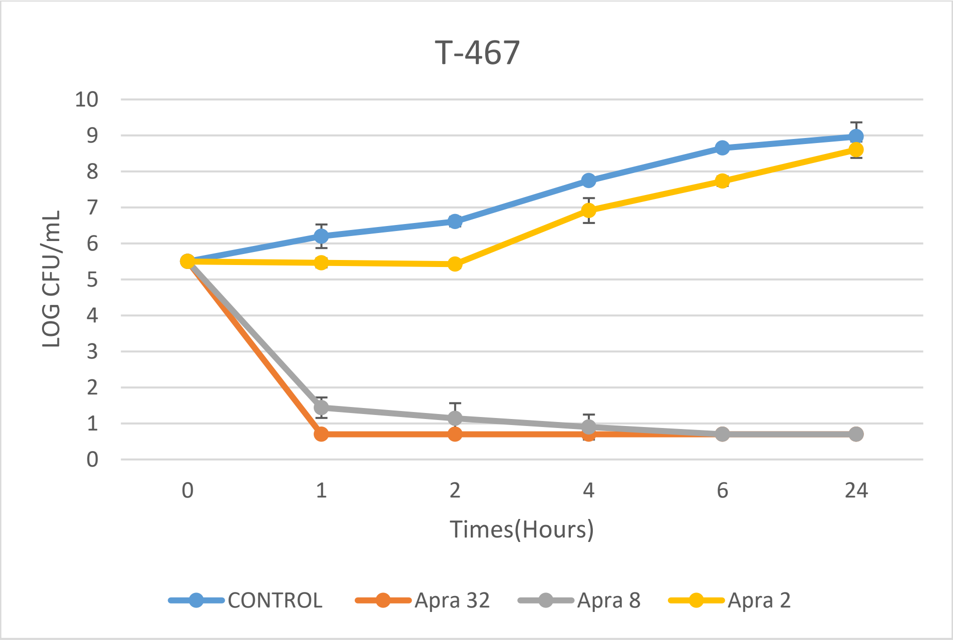

Supplementary Material - Figure 10. Time Kill Kinetics (TKK) of *K. pneumoniae* T-467 using free apramycin (Apra) at concentrations of 2, 8 and 32 mg/L. Shown here are the means of triplicate experiments with error bars indicating the standard deviation.

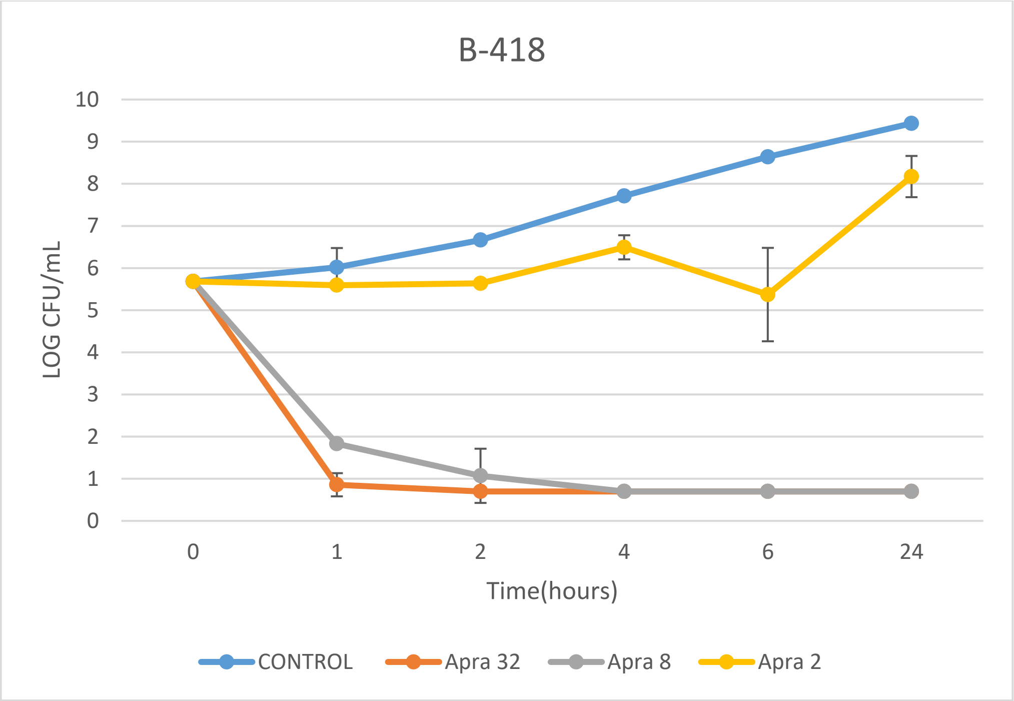

Supplementary Material - Figure 11. Time Kill Kinetics (TKK) of *K. pneumoniae* U-418 using free apramycin (Apra) at concentrations of 2, 8 and 32 mg/L. Shown here are the means of triplicate experiments with error bars indicating the standard deviation.

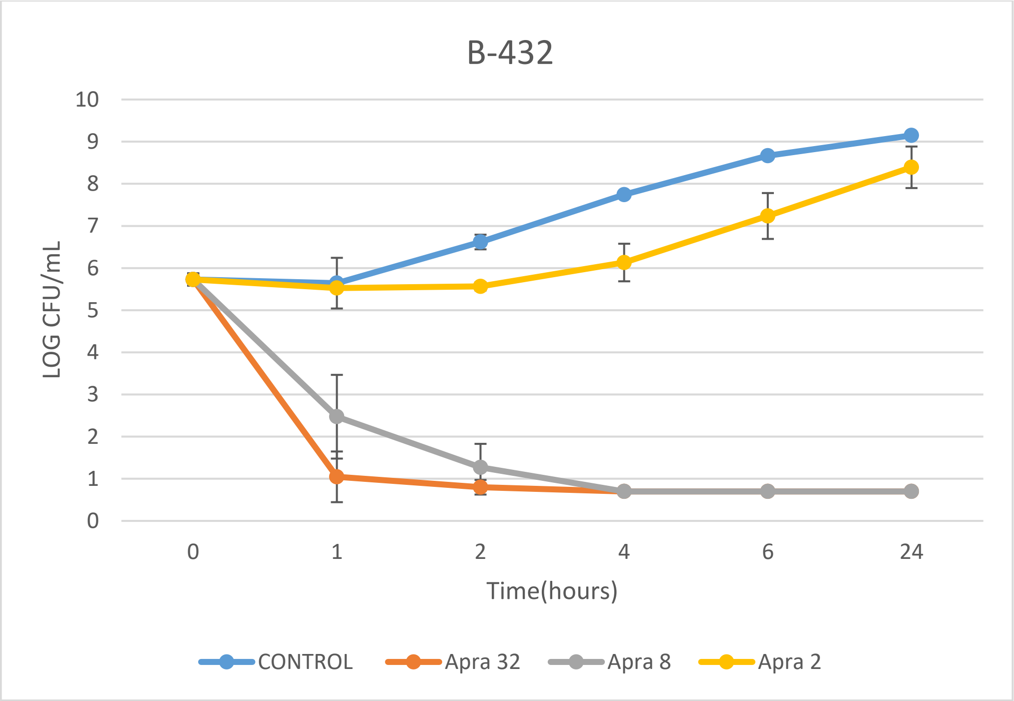

Supplementary Material - Figure 12. Time Kill Kinetics (TKK) of *K. pneumoniae* B-432 using free apramycin (Apra) at concentrations of 2, 8 and 32 mg/L. Shown here are the means of triplicate experiments with error bars indicating the standard deviation.

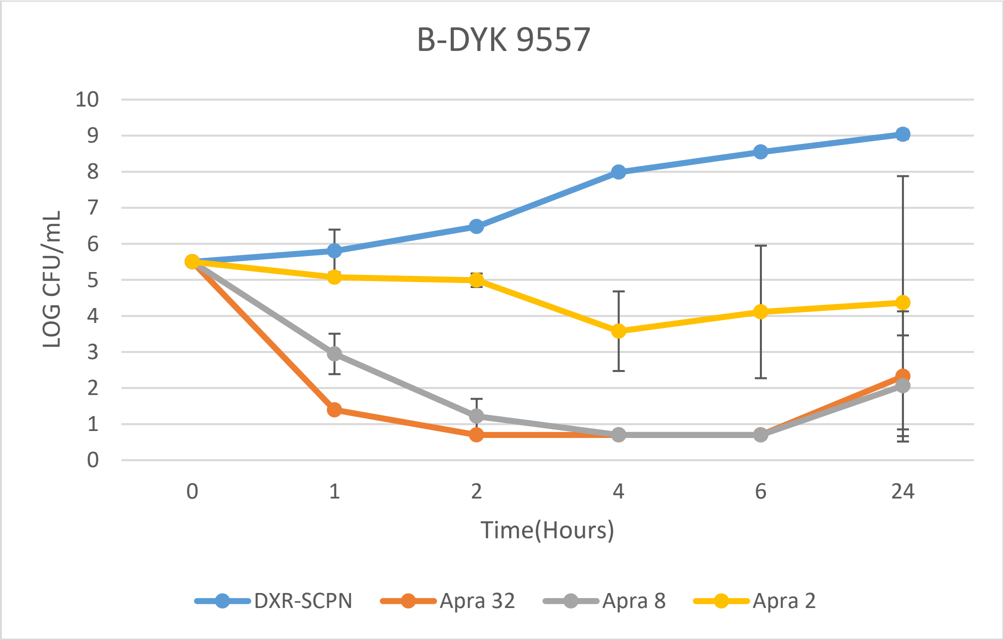

Supplementary Material - Figure 13. Time Kill Kinetics (TKK) of *K. pneumoniae* B-DYK 9557 using DXT-SCPN-Apra at concentrations of 2, 8 and 32 mg/L. Shown here are the means of triplicate experiments with error bars indicating the standard deviation.

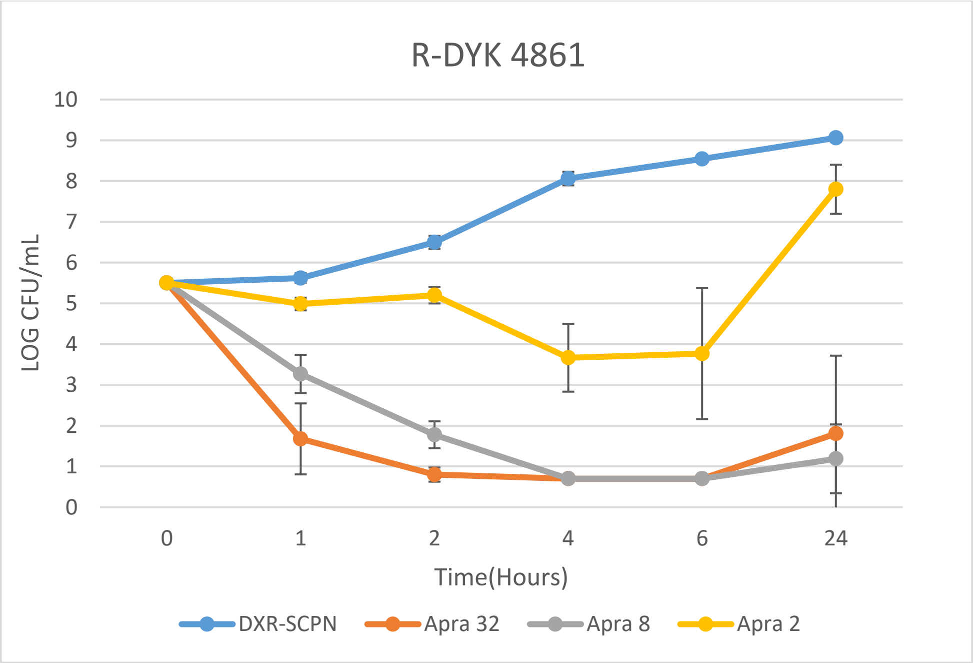

Supplementary Material - Figure 14. Time Kill Kinetics (TKK) of *K. pneumoniae* R-DYK 4861 using DXT-SCPN-Apra at concentrations of 2, 8 and 32 mg/L. Shown here are the means of triplicate experiments with error bars indicating the standard deviation.

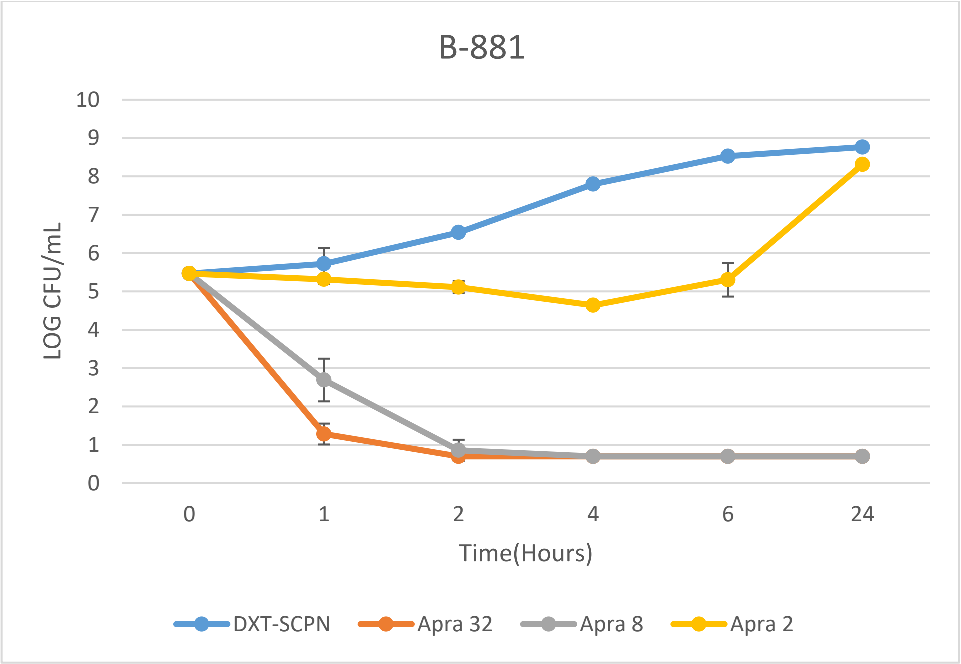

Supplementary Material - Figure 15. Time Kill Kinetics (TKK) of *K. pneumoniae* B-881 using DXT-SCPN-Apra at concentrations of 2, 8 and 32 mg/L. Shown here are the means of triplicate experiments with error bars indicating the standard deviation.

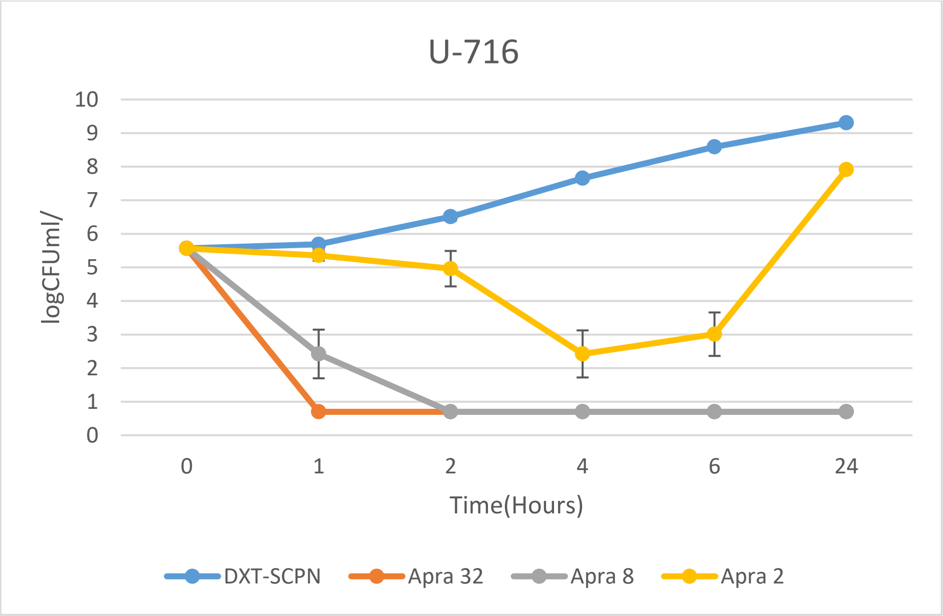

Supplementary Material - Figure 16. Time Kill Kinetics (TKK) of *K. pneumoniae* U-716 using DXT-SCPN-Apra at concentrations of 2, 8 and 32 mg/L. Shown here are the means of triplicate experiments with error bars indicating the standard deviation.

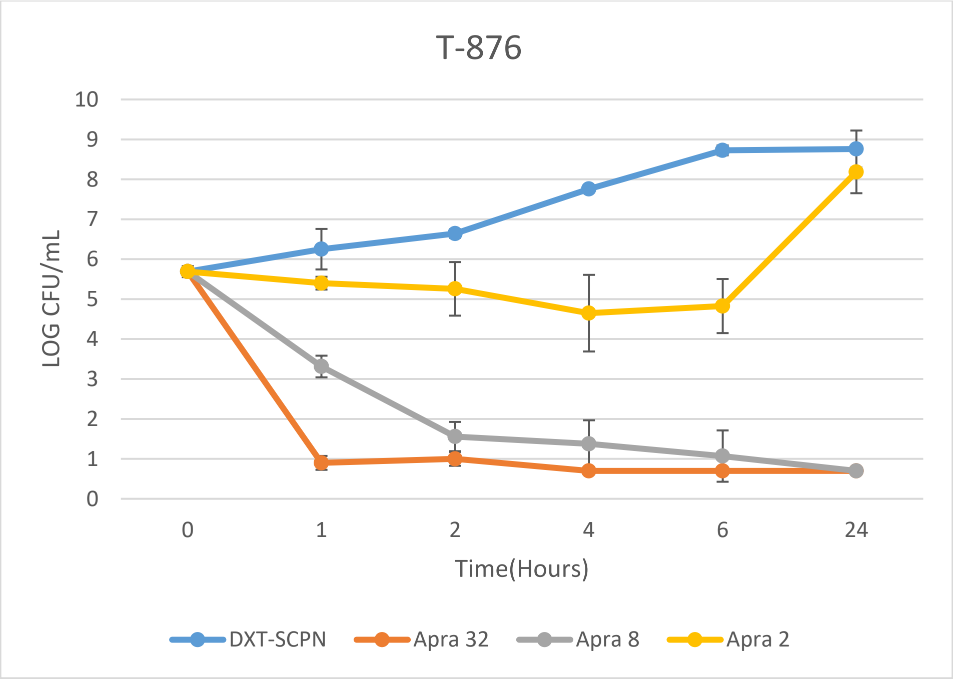

Supplementary Material - Figure 17. Time Kill Kinetics (TKK) of *K. pneumoniae* T-876 using DXT-SCPN-Apra at concentrations of 2, 8 and 32 mg/L. Shown here are the means of triplicate experiments with error bars indicating the standard deviation.

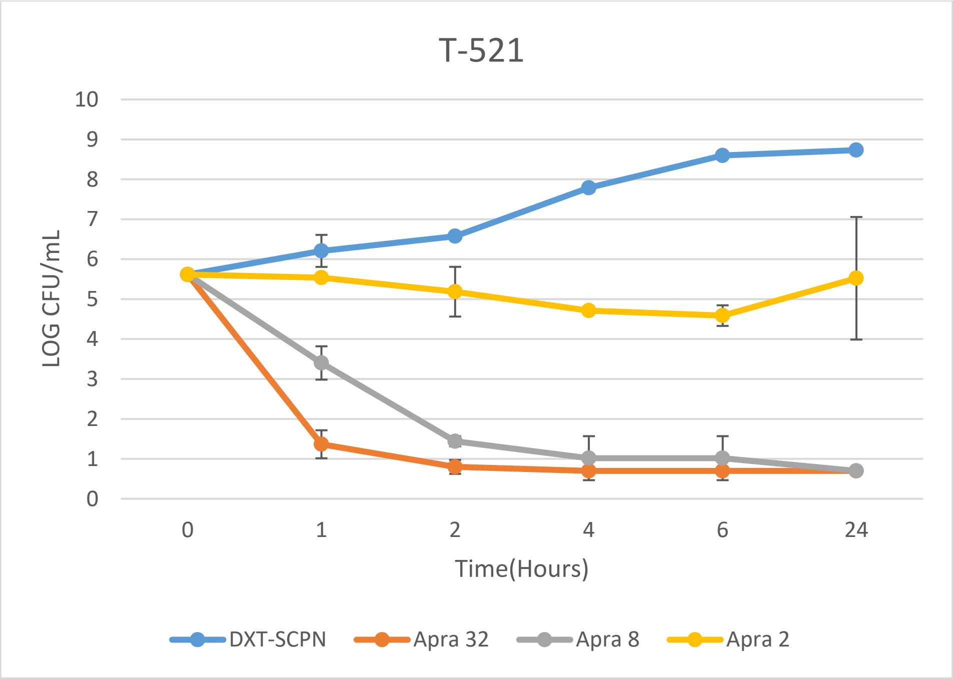

Supplementary Material - Figure 18. Time Kill Kinetics (TKK) of *K. pneumoniae* T-521using DXT-SCPN-Apra at concentrations of 2, 8 and 32 mg/L. Shown here are the means of triplicate experiments with error bars indicating the standard deviation.

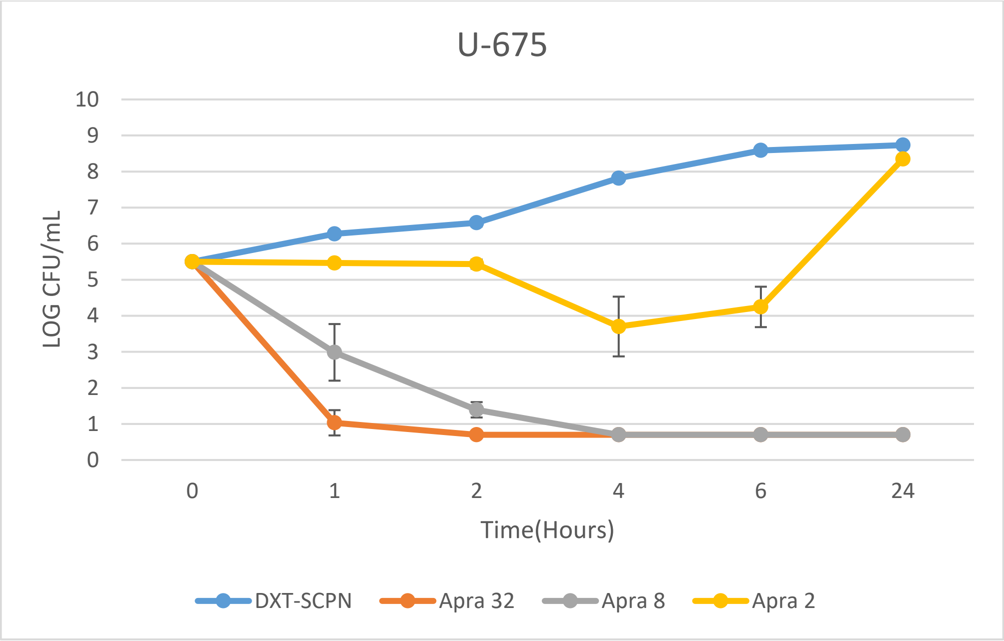

Supplementary Material - Figure 19. Time Kill Kinetics (TKK) of *K. pneumoniae* U-675 using DXT-SCPN-Apra at concentrations of 2, 8 and 32 mg/L. Shown here are the means of triplicate experiments with error bars indicating the standard deviation.

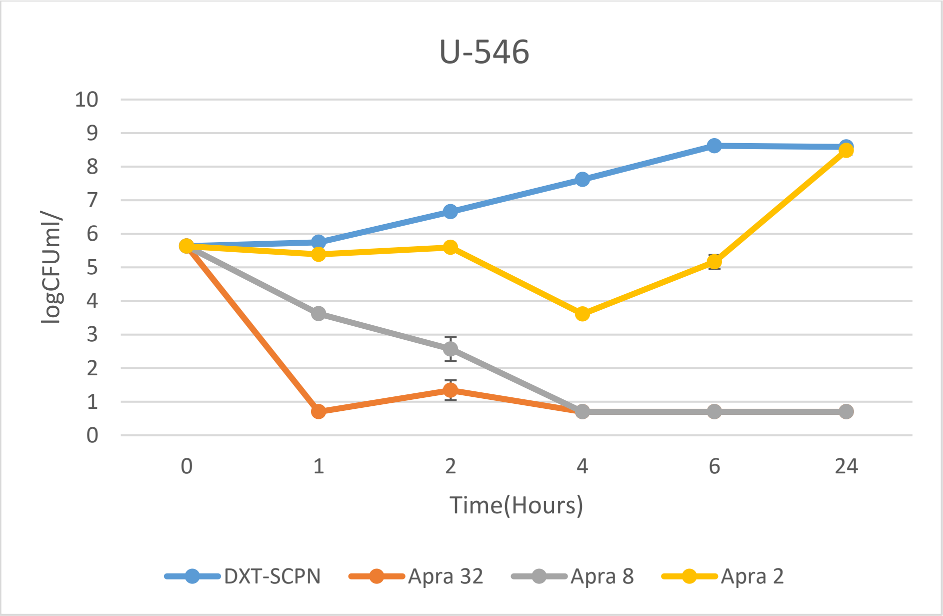

Supplementary Material - Figure 20. Time Kill Kinetics (TKK) of *K. pneumoniae* U-546 using DXT-SCPN-Apra at concentrations of 2, 8 and 32 mg/L. Shown here are the means of triplicate experiments with error bars indicating the standard deviation.

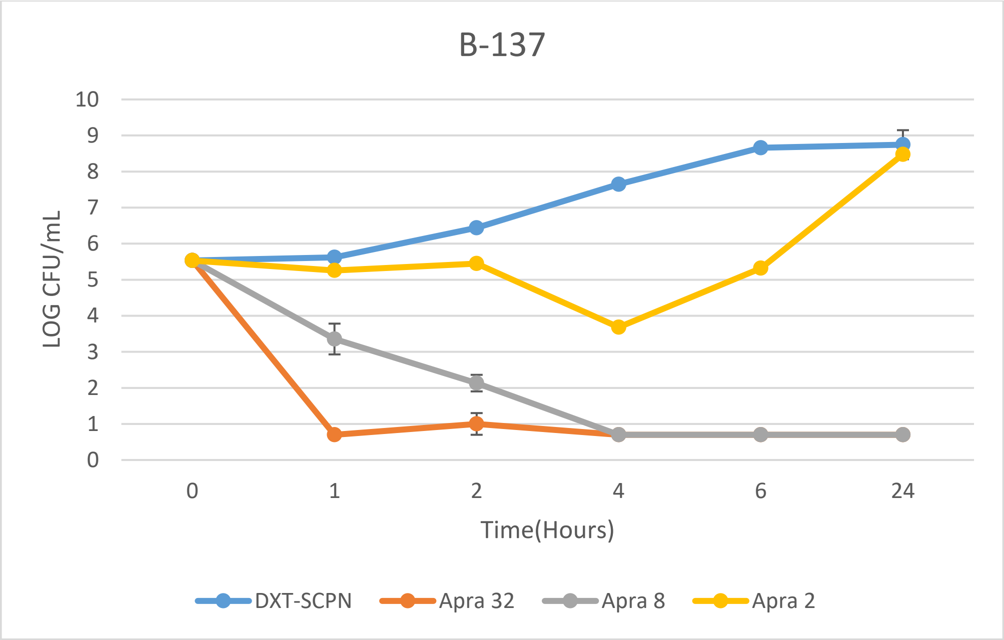

Supplementary Material - Figure 21. Time Kill Kinetics (TKK) of *K. pneumoniae* B-137 using DXT-SCPN-Apra at concentrations of 2, 8 and 32 mg/L. Shown here are the means of triplicate experiments with error bars indicating the standard deviation.

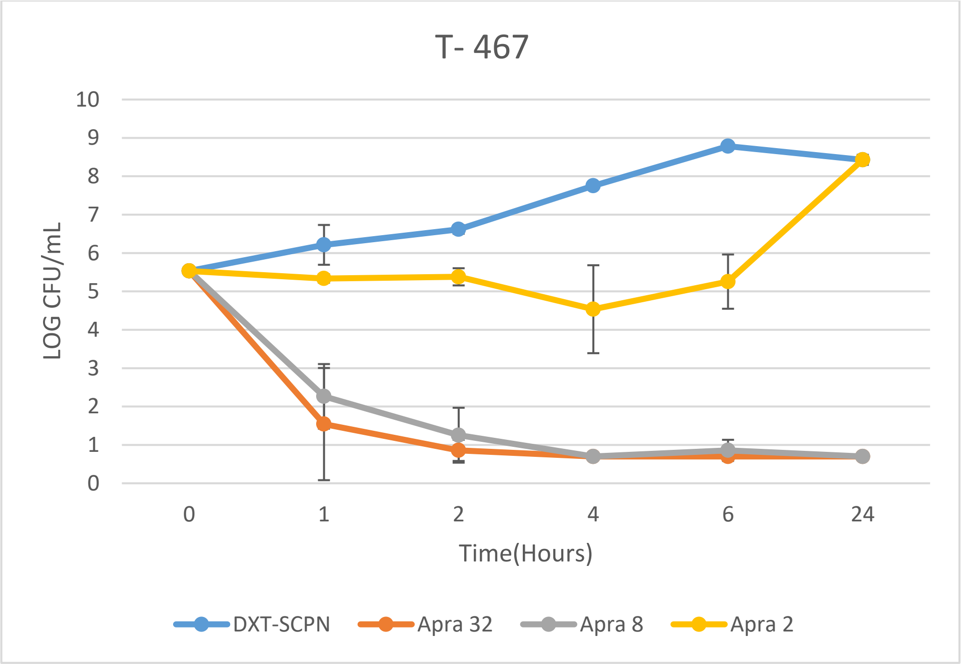

Supplementary Material - Figure 22. Time Kill Kinetics (TKK) of *K. pneumoniae* T-467 using DXT-SCPN-Apra at concentrations of 2, 8 and 32 mg/L. Shown here are the means of triplicate experiments with error bars indicating the standard deviation.

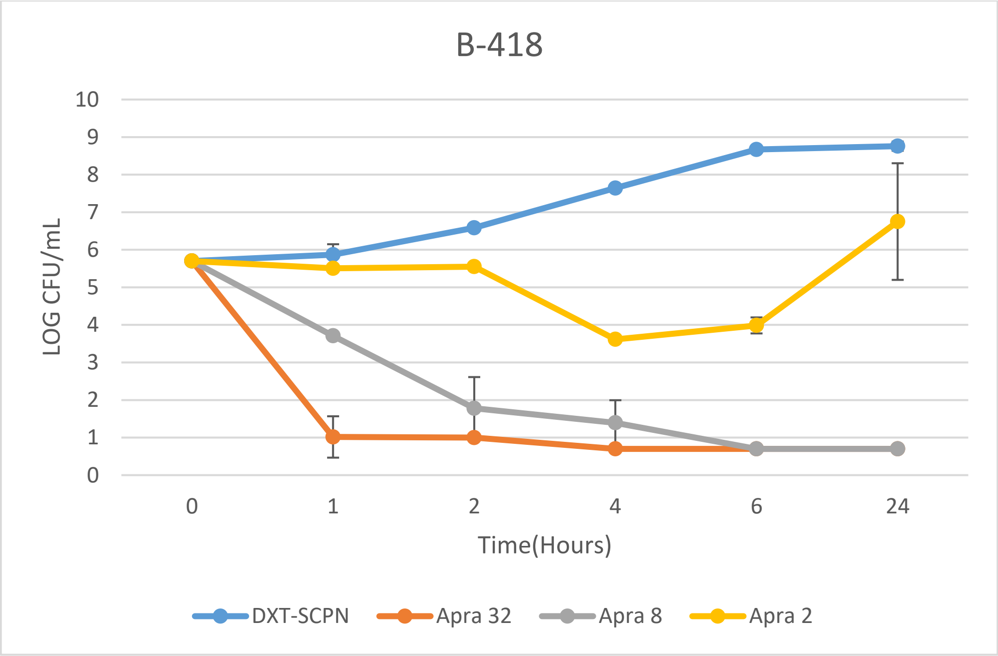

Supplementary Material - Figure 23. Time Kill Kinetics (TKK) of *K. pneumoniae* B-418 using DXT-SCPN-Apra at concentrations of 2, 8 and 32 mg/L. Shown here are the means of triplicate experiments with error bars indicating the standard deviation.

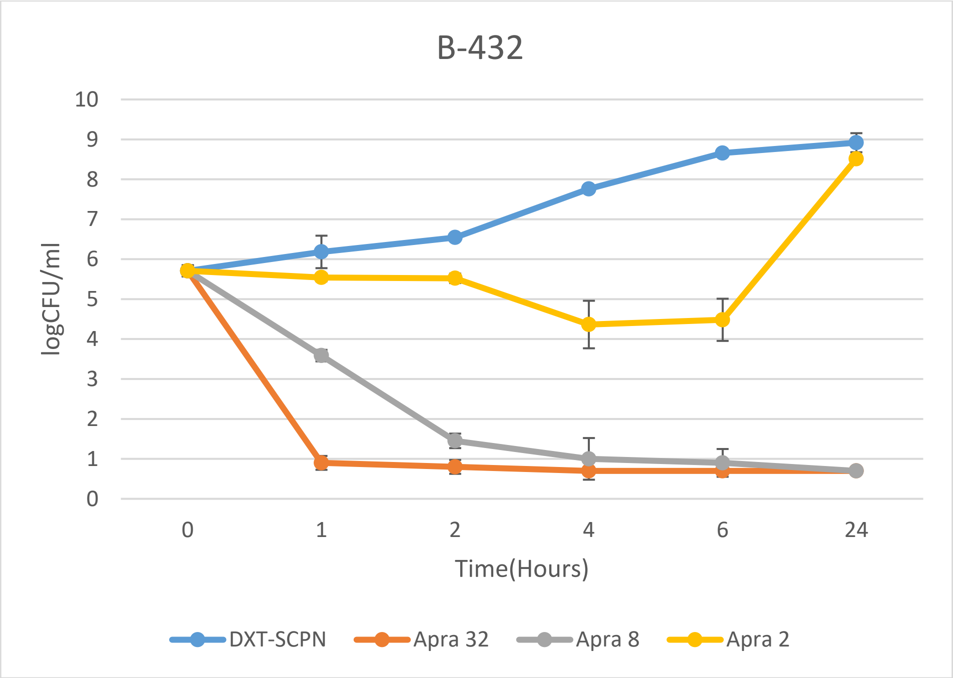

Supplementary Material - Figure 24. Time Kill Kinetics (TKK) of *K. pneumoniae* B-432 using DXT-SCPN-Apra at concentrations of 2, 8 and 32 mg/L. Shown here are the means of triplicate experiments with error bars indicating the standard deviation.

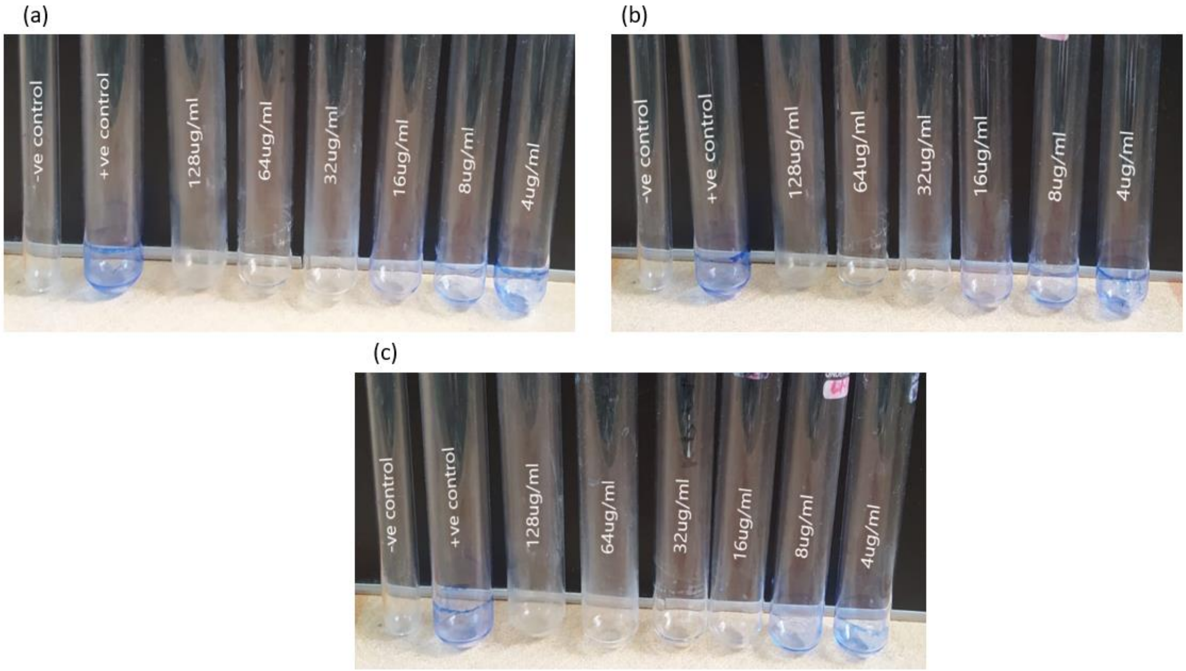

Supplementary Material - Figure 25. Effect of DXT-SCPN-Apra on biofilm formation

Key: (a)=B-881, (b)=T-876 and (c)=U-675

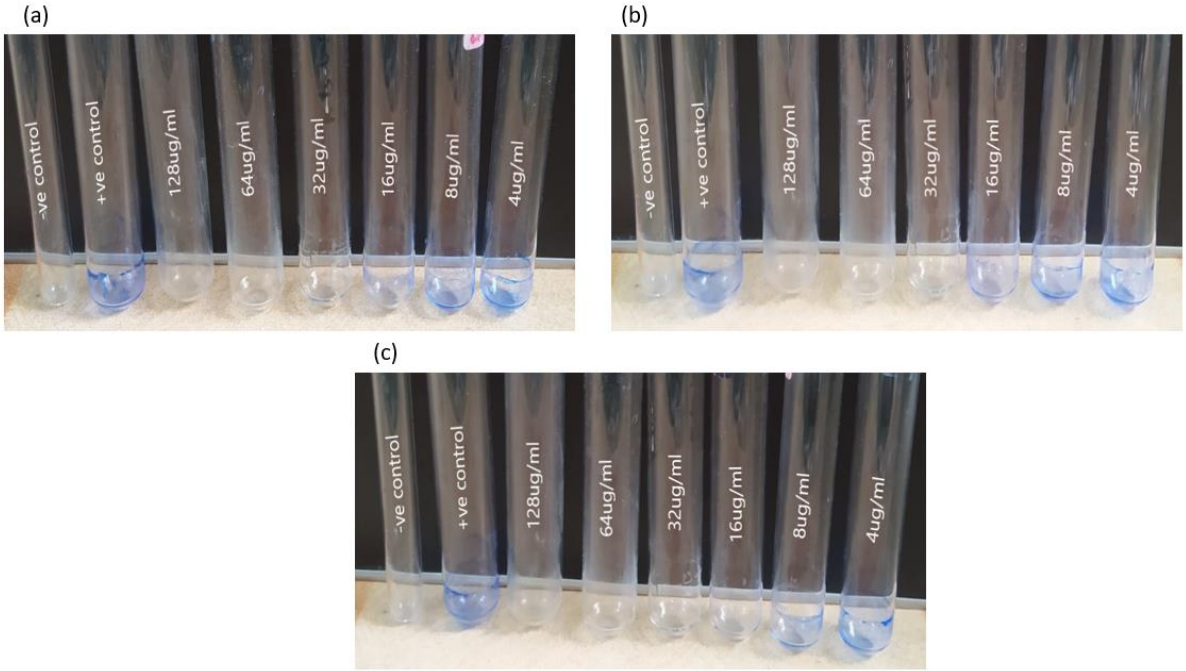

Supplementary Material - Figure 26. Effect of free apramycin (Apra) on biofilm formation

Key: (a)=B-881, (b)=T-876 and (c)=U-675

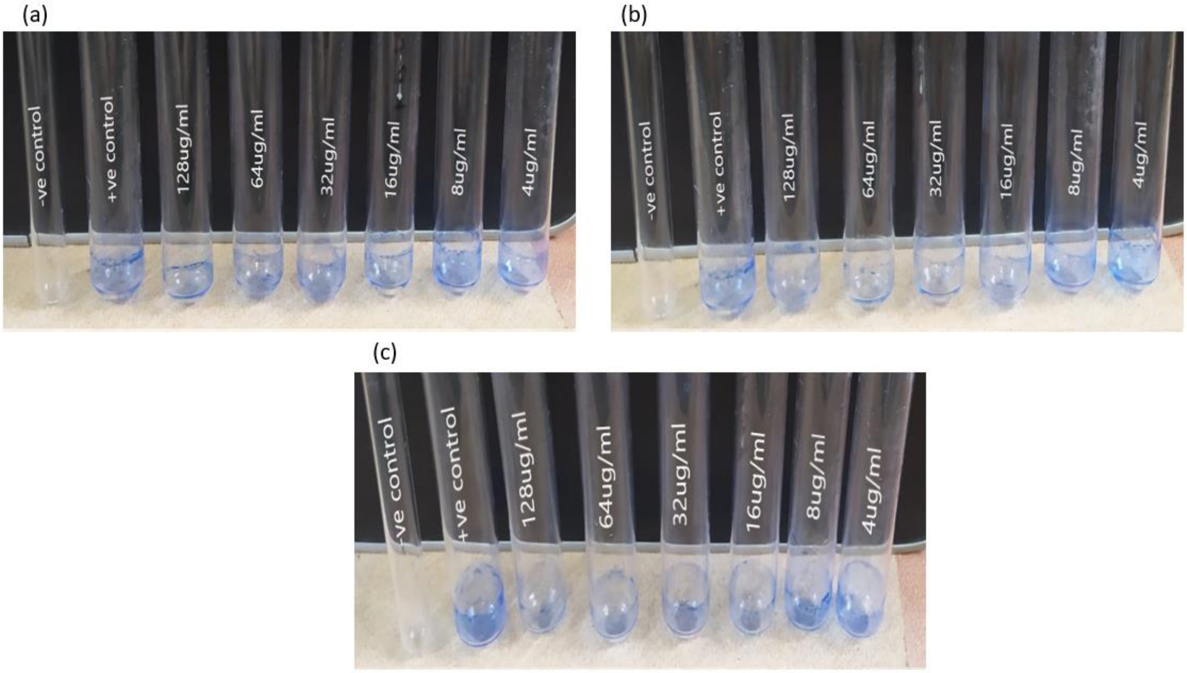

Supplementary Material Figure 27. Effect of empty nanoparticles (DXT-SCPN) on biofilm formation.

Key: (a)=B-81, (b)=T-876 and (c)=U-675
